# SEI1 complex silences p53 to drive myeloma progression

**DOI:** 10.1016/j.isci.2026.117300

**Published:** 2026-08-24

**Authors:** Rui Chen, Rui Liu, Zhihong Fang, Daoyan Yang, Zou Li, Yuan Li, Yuan Li, Shurong Liu, Qi Liu, Yanqi Chao, Chong Wang, Huan Liu

**Affiliations:** 1 Department of Hematology, Xiang’an Hospital of Xiamen University, School of Medicine, Xiamen University, Xiamen 361102, China; 2 Shenzhen Research Institute of Xiamen University, Xiamen 361102, China; 3 Institute for Common Diseases, Jinjiang Municipal Hospital, School of Medicine, Xiamen University, Xiamen 361102, China; 4 Department of Hematology, The First Affiliated Hospital of Xiamen University and Institute of Hematology, School of Medicine, Xiamen University, Xiamen 361102, China; 5 Department of Hematology, The First Affiliated Hospital of Zhengzhou University, Zhengzhou 610054, China

**Keywords:** multiple myeloma, SEI1, p53, transcriptional inactivation

## Abstract

SEI1 is overexpressed in multiple myeloma and correlates with poor prognosis. In this study, we identify SEI1 as a crucial driver of myeloma pathogenesis through a previously unrecognized mechanism: transcriptional inactivation of the tumor suppressor p53. Mechanistically, SEI1 forms a transcriptional complex with histone deacetylase 1 (HDAC1) and forkhead box C1 (FOXC1), which binds to the *p53* promoter, decreases histone H3 lysine 27 acetylation (H3K27ac), and suppresses *p53* transcription, thereby promoting myeloma cell proliferation. SEI1 enhances the stability of the HDAC1-FOXC1 interaction, further amplifying p53 repression. High-throughput virtual screening has identified tiludronate disodium as a potent inhibitor of FOXC1. Tiludronate disodium disrupts the HDAC1-FOXC1 interaction, restores H3K27ac enrichment and p53 expression, and suppresses myeloma growth both *in vitro* and *in vivo*, including a reduction of osteolytic lesions. These findings elucidate the SEI1/HDAC1/FOXC1/p53 axis as a critical driver of myeloma progression and identify tiludronate disodium as a potential therapeutic agent.

## Highlights

• SEI1 forms a complex with HDAC1 and FOXC1 to silence *p53* transcription
• SEI1 enhances HDAC1-FOXC1 interaction, reducing H3K27ac at the *p53* promoter
• Tiludronate disodium disrupts FOXC1-HDAC1, reactivates *p53*, and inhibits myeloma
• The SEI1/HDAC1/FOXC1/p53 axis drives myeloma progression and is therapeutically targetable

## Graphical abstract

**Figure:**
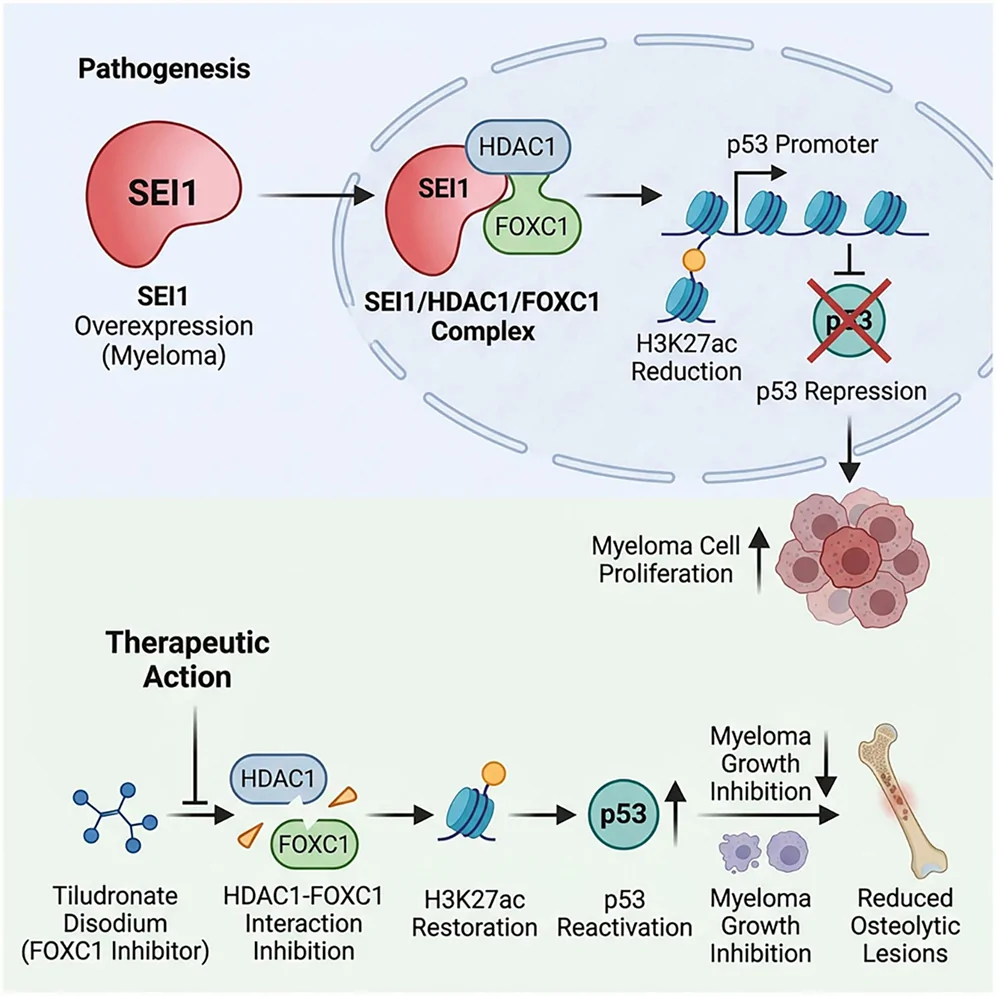

## Teaser

Molecular biology; Epigenetics; Cancer; Omics

## Introduction

Multiple myeloma is an incurable hematologic malignancy originating from plasma cells, representing approximately 10% of all hematologic cancers.^1^ This disease predominantly affects middle-aged and elderly individuals, with its incidence steadily increasing due to population aging, thus making myeloma the second most common hematologic malignancy after lymphoma.^2^ Characterized by the proliferation of malignant plasma cells and infiltration of the bone marrow, along with excessive secretion of monoclonal immunoglobulin, the disease results in clinical manifestations, such as osteolytic lesions, pathological fractures, hypercalcemia, anemia, and hyperviscosity syndrome, ultimately leading to a poor prognosis.^3^

The pathogenesis of myeloma is complex and highly heterogeneous, driven by genetic abnormalities, somatic mutations, bone marrow microenvironment reprogramming, metabolic dysregulation, and epigenetic alterations. Chromosomal translocations are pivotal genetic events in myeloma pathogenesis. Recurrent IgH rearrangements, including t(4;14), t(11;14), t(14;16), and t(14;20), drive aberrant fusion of the immunoglobulin heavy chain locus with oncogenes, such as fibroblast growth factor receptor 3 (FGFR3), cyclin D1, cyclin D2, and mucosa-associated lymphoid tissue lymphoma translocation protein 1 (MALT1). These alterations subsequently upregulate oncogenes like c-MYC, promoting myeloma cell proliferation and survival.^4^^,^^5^^,^^6^^,^^7^ Aneuploidy occurs in ∼50% of myeloma patients, disrupting cellular growth and differentiation to promote tumor initiation and progression.^8^ Somatic mutations in kirsten rat sarcoma viral oncogene homolog (KRAS), p53, and c-MYC frequently dysregulate cell cycle, apoptosis, and signaling, thereby driving malignant transformation.^9^^,^^10^^,^^11^^,^^12^ Metabolic reprogramming is a hallmark of myeloma. These adaptations fuel tumor cell survival and proliferation under hypoxic and nutrient-deprived conditions by supplying energy and biosynthetic intermediates.^13^ Additionally, inactivation of tumor suppressor genes, such as retinoblastoma (Rb) and p53,^14^ activation of the nuclear factor kappa B (NF-κB) pathway,^15^ and aberrant Wnt signaling are commonly implicated in myeloma pathogenesis.^16^ Thus, the multifaceted pathogenesis of myeloma underlies its therapeutic intractability. Elucidating these mechanisms is therefore critical for developing targeted strategies to improve clinical outcomes.

SERTA domain-containing protein 1 (SERTAD1) (also known as SEI1 or TRIP-Br1) is a nuclear oncoprotein of the SERTAD family that modulates the cell cycle via CDK4 and E2F/DP-1.^17^ It drives chromosomal instability, inhibits apoptosis through PI3K-Akt/ILK signaling, and promotes metastasis in HER2/neu-positive and -negative cancer cells.^18^ Chemotherapeutic agents (bortezomib and melphalan) induce DNA damage in myeloma cells, activating the cyclic guanosine monophosphate (GMP)-adenosine monophosphate (AMP) synthase (cGAS)/stimulator of interferon genes (STING) pathway and interferon regulatory factor 7 (IRF7) phosphorylation, which in turn upregulates SEI1 transcription. Elevated SEI1 interacts with the CREB-binding protein (CBP)/p300 and the RNA polymerase II (pol II)-associated factor 1 (PAF1) complex to enhance programmed death ligand-1 (PD-L1) expression through super-enhancer amplification, thereby facilitating immune evasion.^19^ However, it remains unclear whether SEI1 directly regulates malignant progression in myeloma.

Using *in vitro*, *in vivo*, and patient sample studies, we identify SEI1 as a driver of myeloma progression. SEI1 is overexpressed in myeloma and forms a complex with HDAC1 and FOXC1 that suppresses p53 transcription by reducing H3K27ac at the *p53* promoter, accelerating myeloma cell proliferation. SEI1 also stabilizes the HDAC1-FOXC1 interaction, amplifying *p53* repression. High-throughput screening reveals tiludronate disodium as a FOXC1 inhibitor that disrupts this interaction, restores *p53* transcription, and inhibits myeloma growth. Our findings define SEI1/HDAC1/FOXC1/p53 axis in myeloma pathogenesis and establish SEI1 as a therapeutic target, offering a strategy against this incurable disease.

## Results

### SEI1 is critical for myeloma disease progression

**Figure 1:**
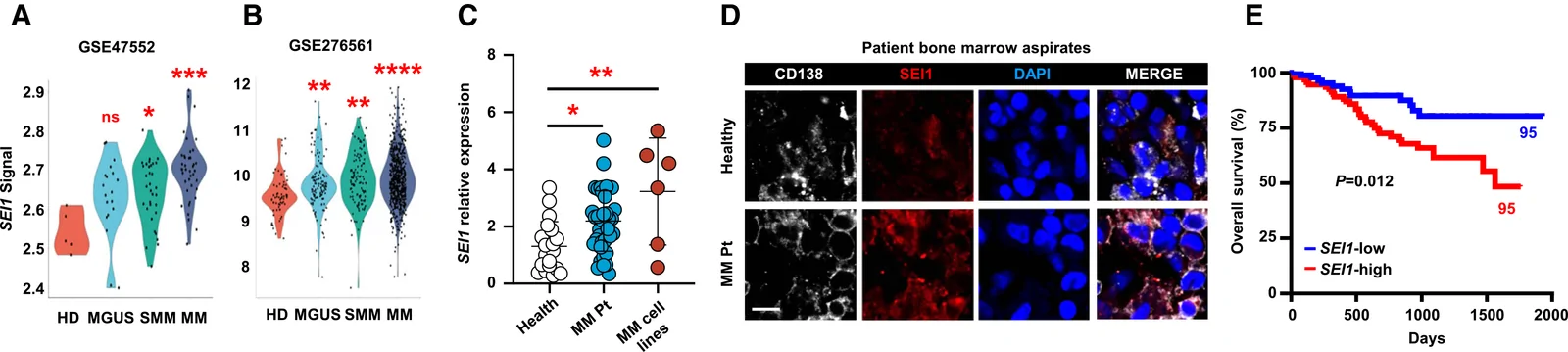
SEI1 is critical for myeloma disease progression (A) *SEI1* mRNA levels in plasma cells from myeloma patients (MM, *n* = 41), smoldering multiple myeloma (sMM, *n* = 33), or monoclonal gammopathy of undetermined significance (MGUS, *n* = 20) compared to normal plasma cells from healthy donors (HD, *n* = 5) (GEO: GSE47552). (B) *SEI1* mRNA levels in plasma cells from myeloma patients (MM, *n* = 197), smoldering multiple myeloma (sMM, *n* = 122), or monoclonal gammopathy of undetermined significance (MGUS, *n* = 122) compared to normal plasma cells from healthy donors (HD, *n* = 67) (GEO: GSE276561). (C) Quantitative real-time PCR analysis shows *SEI1* expression in normal plasma cells (Health, *n* = 20), malignant plasma cells (MM Pt, *n* = 20), and human myeloma cell lines (MM cell lines, *n* = 6). GAPDH served as loading control. (A–C) *p* values were determined using one-way ANOVA. ns, not significant. (D) Immunofluorescent staining of healthy of myeloma patients bone marrow samples with DAPI and antibodies against CD138 and SEI1 (*n* = 5). Scale bars, 10 μm. (E) Overall survival (OS) of patient’s myeloma cells with high SEI1 (High, *n* = 95) and low SEI1 (Low, *n* = 95) expression in MMRF CoMMpass study IA15. Data are averages ±SD. ∗*p* < 0.05, ∗∗*p* < 0.01, ∗∗∗*p* < 0.001, ∗∗∗∗*p* < 0.0001.

By utilizing the Gene Expression Omnibus (GEO) database (GSE124610), published single-cell sequencing data indicated that myeloma cells (CD138^+^ cells) exhibit elevated levels of SEI1 (Figure S1). We further analyzed the expression dynamics of *SEI1* during myeloma progression. The results demonstrated that *SEI1* expression progressively increases with disease advancement (GSE47552 and GSE276561) (Figures 1A and 1B), underscoring its critical role in myeloma progression. Quantitative real-time PCR results revealed that *SEI1* expression was elevated in the majority of bone marrow samples obtained from primary myeloma cells and human myeloma cell lines compared to normal plasma cells (Figure 1C). Additionally, immunofluorescence analysis of bone marrow samples from myeloma patients showed increased SEI1 expression of SEI1 in myeloma cells (Figure 1D). In our subsequent analysis of the relationship between SEI1 expression and myeloma progression, utilizing data from the Multiple Myeloma Research Foundation (MMRF) CoMMpass study IA15, we found that patients with high SEI1 expression in myeloma cells exhibited a lower overall survival rate compared to those with low expression levels (Figure 1E).

**Figure 2:**
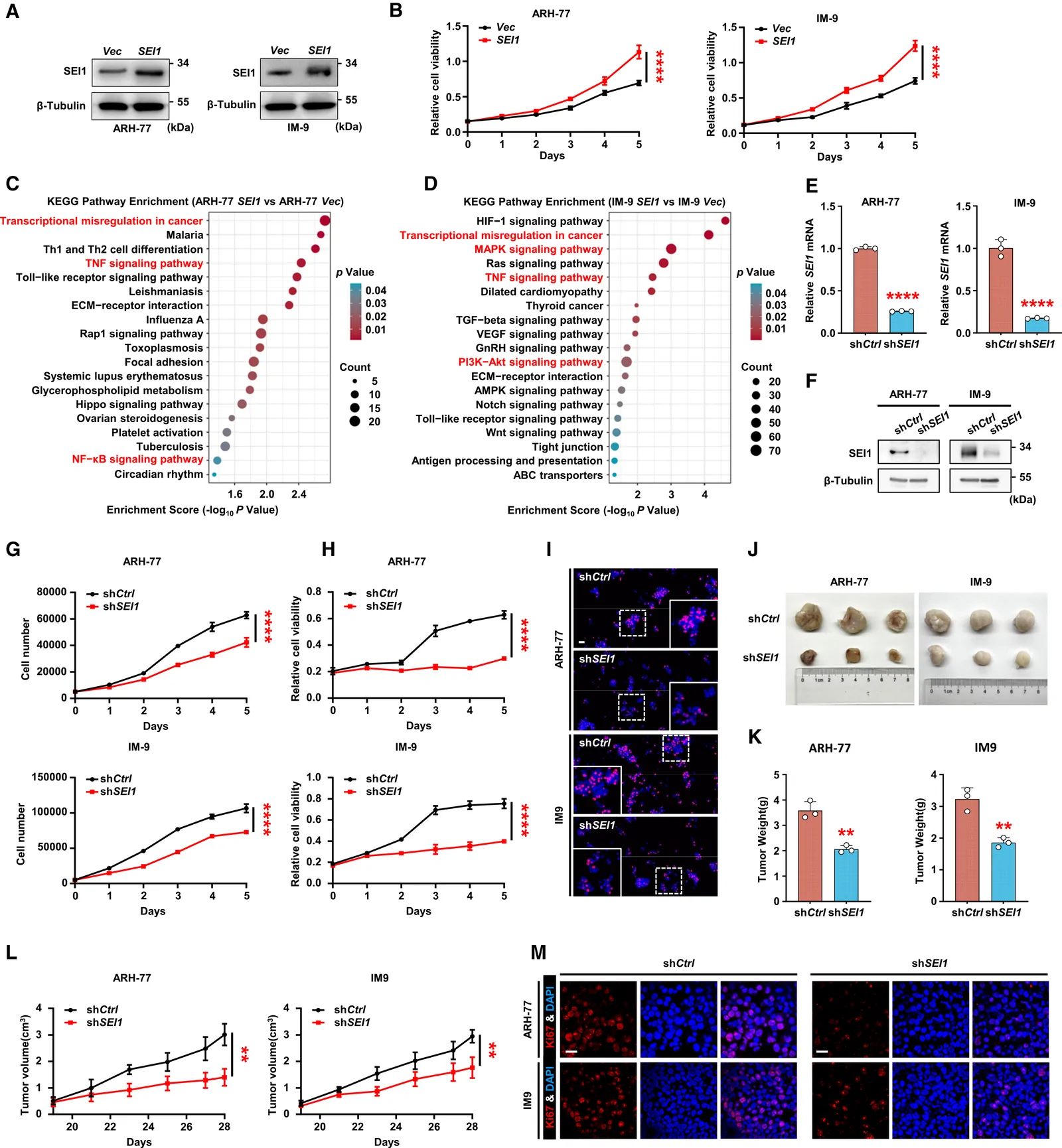
SEI1 promotes growth of myeloma cells (A) Western blot analysis shows the expression of SEI1-overexpressing myeloma cell lines (ARH-77 or IM-9) compared to the vector control. (B) Proliferation of myeloma cells with increased (*SEI1*) SEI1 expression over time. Vector control (*Vec*) served as control. (C and D) RNA-seq analysis of pathway enrichment in SEI1-overexpressing myeloma cells (*SEI1*) compared to control cells (*Vec*). (E and F) Quantitative real-time PCR and western blot analysis shows the downregulation of SEI1 in knockdown cell lines (ARH-77 or IM-9) compared to the shRNA control. (G and H) Proliferation of myeloma cells with reduced (sh*SEI1*) SEI1 expression over time. Nontargeted shRNA (sh*Ctrl*) served as control. (I) Representative images and the percentage of EdU-positive cells of ARH-77 or IM-9 cells (sh*Ctrl* or sh*SEI1*). Scale bars, 40 μm. (*n* = 3 biological replicates). (J) Photographic images of tumors in NSG mice after implantation of ARH-77 or IM-9 (sh*Ctrl* or sh*SEI1*) cells (*n* = 3 mice/group). (K and L) Tumor weight and time course of tumor volume. (M) Immunofluorescent staining of Ki67 expression in tumor tissues (*n* = 3 mice/group). Scale bars, 20 μm. (A), (B), and (E–I) are representative of three independent experiments. Data are averages ±SD. ∗∗*p* < 0.01, ∗∗∗∗*p* < 0.0001. (E) and (K): *p* values were determined by unpaired two-tailed *t* test; (B), (G), (H), and (L): *p* values were determined using two-way ANOVA.

To elucidate the role of SEI1 in oncogenic processes, we introduced external SEI1 cDNA into ARH-77 or IM-9 cells to achieve overexpression (Figure 2A). Cell proliferation assays showed that ectopic overexpression of SEI1 significantly promoted the proliferation of myeloma cells compared to the vector control group (Figure 2B). Subsequently, we performed RNA sequencing (RNA-seq) transcriptome analysis to investigate SEI1 overexpression altered transcription in myeloma cells compared to control cells. Using the Broad Institute’s Molecular Signatures Database (MSD) hallmark gene sets for computational analysis, we observed broad enrichment of genes associated with cell proliferation, like those tied to transcriptional misregulation in cancer, TNF, MAPK, PI3K-Akt, and NF-κB signaling pathways, were broadly enriched (Figures 2C and 2D). Additionally, we used small hairpin RNAs (shRNAs) to silence SEI1 in ARH-77 and IM-9 myeloma cell lines (Figures 2E and 2F). In cell growth assays, myeloma cells with elevated SEI1 levels exhibited accelerated growth than their SEI1-depleted counterparts (Figures 2G and 2H). Consistent with these findings, EdU assays indicated that myeloma cells with SEI1 knockdown exhibited reduced colony-forming capability compared to control cells (Figure 2I). To further validate our observations, we subcutaneously injected ARH-77 or IM-9 cells (sh*Ctrl* and sh*SEI1*) into NSG (NOD scid gamma) mice. The results showed that SEI1 suppression significantly slowed tumor progression in recipient mice compared to control group (Figures 2J–2M). In addition, to determine whether the growth inhibition observed upon SEI1 is due to apoptosis or cell-cycle arrest, we have performed flow cytometry-based apoptosis and cell-cycle assays in SEI1 knockdown myeloma cells. As shown in Figures S2A and S2B, SEI1 knockdown significantly increased the percentage of apoptotic cells compared to the control group. Furthermore, cell-cycle analysis revealed that SEI1 knockdown induced a prominent G1-phase arrest, accompanied by a reduced S-phase population (Figures S2A and S2B). Overall, these findings highlight the role of SEI1 in promoting myeloma cell proliferation.

### SEI1 forms a complex with HDAC1 and FOXC1 to inhibit *p53* transcription

**Figure 3:**
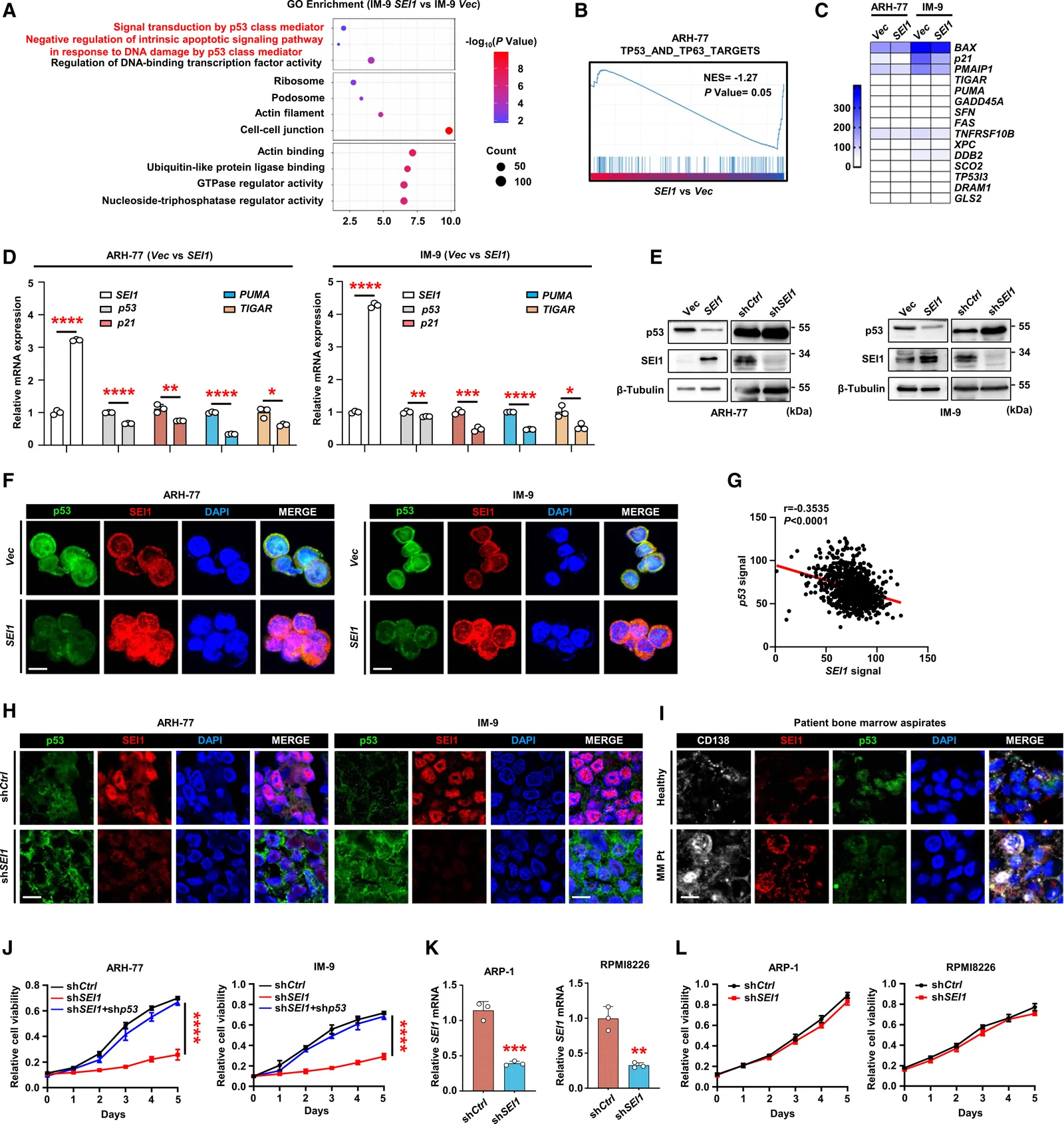
SEI1 inhibits *p53* transcription (A) RNA-seq analysis of pathway enrichment in SEI1-overexpressing myeloma cells (*SEI1*) compared to control cells (*Vec*). (B) GSEA analysis of RNA-seq datasets in Figure 2 of the p53 target genes in SEI1-overexpressing myeloma cells (*SEI1*) compared to control cells (*Vec*). (C) Heatmap showing the expression profile of p53 target genes in SEI1-overexpressing myeloma cells (*SEI1*) compared to control cells (*Vec*). (D) Shown are the expression of *SEI1*, *p53*, *p21*, *PUMA*, and *TIGAR* in SEI1-overexpressing myeloma cells (*SEI1*) compared to control cells (*Vec*). (E) The expression of SEI1 or p53 in myeloma cells with reduced (sh*SEI1*) or increased (*SEI1*) SEI1 expression. Nontargeted shRNA (sh*Ctrl*) or control vector (*Vec*) served as control. (F) Immunofluorescent staining of ARH-77 or IM-9 cells (*Vec* and *SEI1*) with DAPI and antibody against p53 and SEI1. Scale bars, 10 μm. (G) Correlation coefficient of the mRNA levels of *SEI1* and mRNA levels of *p53* in myeloma patients (*n* = 859). The correlations were evaluated using Pearson coefficient. r, correlation coefficient. (H) Immunofluorescent staining of tumor tissues in Figure 2J with DAPI and antibodies against p53 and SEI1 (*n* = 3 mice/group). Scale bars, 10 μm. (I) Immunofluorescent staining of myeloma patients bone marrow samples with DAPI and antibodies against CD138, SEI1 and p53 (*n* = 5). Scale bar, 10 μm. (J) Proliferation of myeloma cells (ARH-77 or IM9) with reduced SEI1 expression (sh*SEI1*) or with reduced expression of both SEI1 and p53 (sh*SEI1*+sh*p53*) over time. Nontargeted shRNA (sh*Ctrl*) served as control. (K) Quantitative real-time PCR analysis shows the downregulation of SEI1 in knockdown cell lines (ARP-1 or RPMI8226) compared to the shRNA control. (L) Proliferation of myeloma cells (ARP-1 or RPMI8226) with reduced SEI1 expression (sh*SEI1*) over time. (D–F) and (J–L) are representative of three independent experiments. Data are averages ±SD. ∗*p* < 0.05, ∗∗*p* < 0.01, ∗∗∗*p* < 0.001, ∗∗∗∗*p* < 0.0001. (D) and (K): *p* values were determined by unpaired two-tailed *t* test; (J) and (L): *p* values were determined using two-way ANOVA.

To further explore the mechanism by which SEI1 regulates myeloma cell proliferation, we analyzed RNA-seq data from SEI1-overexpressing myeloma cell lines versus control cells with Gene Ontology enrichment analysis (GO enrichment). Among the hallmark gene sets, the p53 pathway was the most significantly negatively enriched in SEI1-overexpressing cells (Figure 3A), prompting us to investigate whether SEI1 suppresses p53 transcription as a key downstream mechanism. Moreover, through gene set enrichment analysis (GSEA) and differential gene expression analysis, we also identified significant downregulation of p53 pathway target genes in SEI1-overexpressing cells (Figures 3B and 3C). Quantitative real-time PCR results revealed that SEI1 overexpression in myeloma cells inhibited the transcription of p53 and the expression of its downstream genes (*p21*, *PUMA*, and *TIGAR*) (Figure 3D). Additionally, western blot experiments also demonstrated that SEI1 suppresses p53 expression (Figure 3E). Furthermore, immunofluorescence analysis also confirmed that SEI1 suppresses p53 expression (Figure 3F). Correlation analysis of primary myeloma cells indicated a negative correlation between *SEI1* expression and *p53* mRNA levels (Figure 3G). Immunofluorescence analysis of subcutaneous xenograft tumors in mice further demonstrated that SEI1 knockdown in myeloma cells promotes p53 expression (Figure 3H). Moreover, immunofluorescence analysis of bone marrow samples from myeloma patients indicated that SEI1 represses p53 transcription in myeloma cells (Figure 3I). Collectively, these findings demonstrate that SEI1 represses p53 transcription in myeloma cells.

To test whether SEI1-induced myeloma cell growth is specifically mediated by p53 suppression, we performed either SEI1 knockdown alone or combined SEI1 and p53 knockdown in IM-9 and ARH-77 cells. Knockdown of p53 significantly reversed the inhibitory effect of SEI1 knockdown on myeloma cell proliferation (Figure 3J). We further conducted SEI1 knockdown experiments in myeloma cell lines lacking functional p53, including ARP1 (p53 null) and RPMI8226 (p53 mutated) cells. Although SEI1 knockdown effectively reduced SEI1 expression in both cell lines (Figure 3K), it did not significantly inhibit their proliferation (Figure 3L), in stark contrast to the pronounced growth-suppressive effects observed in p53-wild-type myeloma cells (Figure 3J). Collectively, these results demonstrate that the growth-inhibitory effect of SEI1 knockdown is largely dependent on the presence of functional p53.

**Figure 4:**
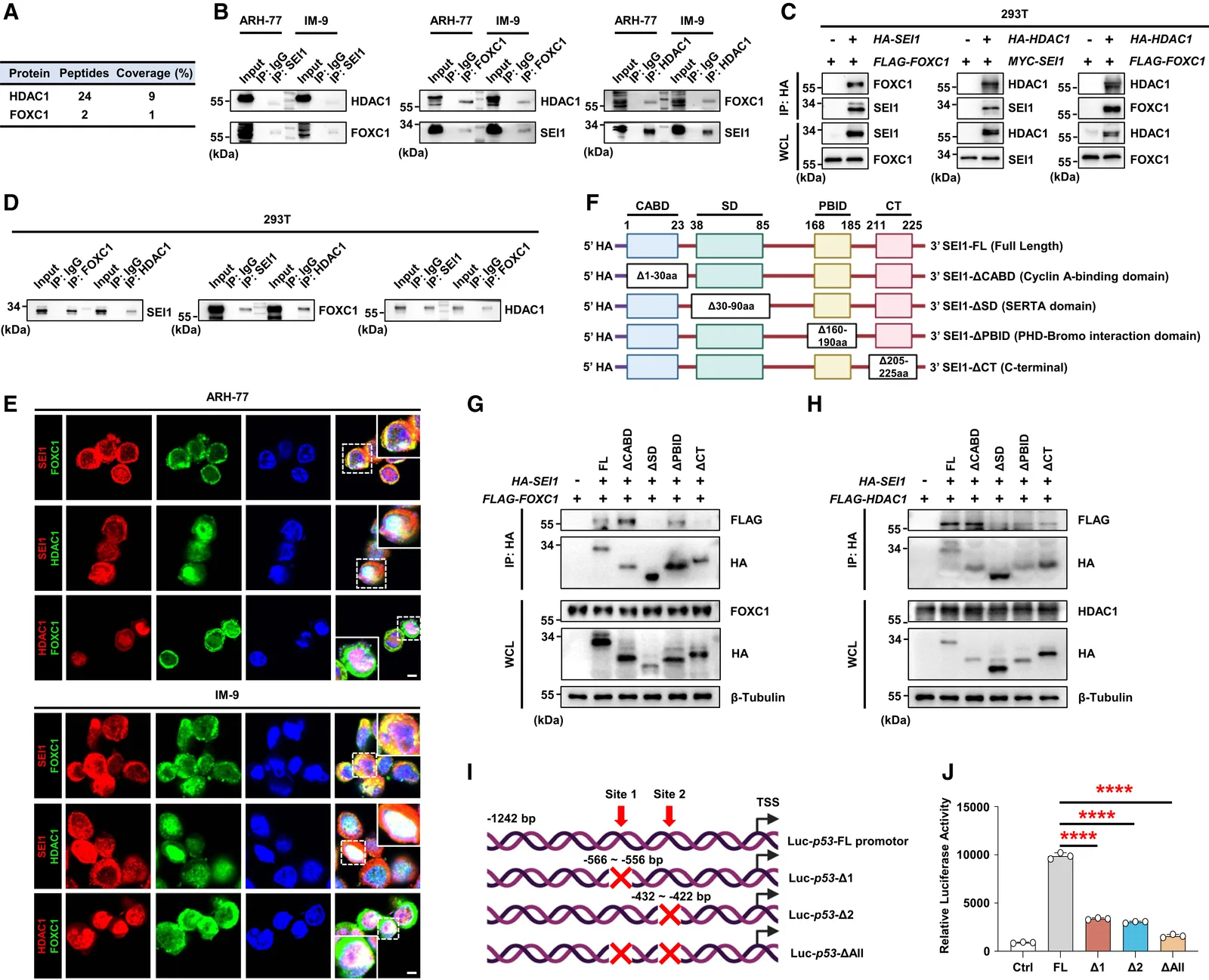
SEI1 forms a complex with HDAC1 and FOXC1 to inhibit *p53* transcription (A) HA-SEI1 overexpressing IM-9 cells were lysed. Immunoprecipitation of the resulting total protein lysate was performed utilizing agarose-immobilized HA antibody, followed by analysis via mass spectrometry. The identified proteins are shown. (B) Co-immunoprecipitation of SEI1, HDAC1, or FOXC1 in ARH-77 or IM-9 cells. (C) Pull down of HA-SEI1 with FLAG-FOXC1, HA-HDAC1 with MYC-SEI1, or FLAG-FOXC1 in HEK293T cells. (D) Co-immunoprecipitation assays were performed using HEK293T cells co-transfected with *SEI1* and *HDAC1* plasmids, *SEI1* and *FOXC1* plasmids, or *HDAC1* and *FOXC1* plasmids, to evaluate the interactions among SEI1, HDAC1, and FOXC1. (E) Immunofluorescent staining of ARH-77 or IM-9 cells with DAPI, anti-SEI1 and anti-FOXC1 antibodies, or anti-HDAC1 and anti-FOXC1 antibodies. Scale bars, 5 μm. (F) Schematic of SEI1 protein truncations, including ΔCABD, ΔSD, ΔPBID, and ΔCT fragments. (G and H) Pull down of FOXC1 or HDAC1 with different truncations of SEI1 (Full length, ΔCABD, ΔSD, ΔPBID, and ΔCT) in HEK293T cells. (I and J) Schematic of the *p53* promoter luciferase reporter. Rred crosses: mutations of nucleotides. The luciferase activity of Luc-*p53* constructs was set at 1. (B–E), (G), (H), and (J) are representative of three independent experiments. Data are averages ±SD. ∗∗∗∗*p* < 0.0001. (J): *p* values were determined using one way ANOVA.

To elucidate the molecular mechanism by which SEI1 regulates p53, we performed immunoprecipitation followed by mass spectrometry analyses. These analyses identified HDAC1 and FOXC1 proteins as specific binding partners of SEI1 (Figure 4A and Tables S1 and S2). HDAC1 is an enzyme that removes acetyl groups from histones. It helps decrease gene expression by compacting chromatin. HDAC1 is involved in various cellular processes and is a target in cancer therapy research.^20^ The forkhead box transcription factor FOXC1 binds DNA to regulate gene expression, contributing critically to both embryonic development and tumor progression and metastasis.^21^ To investigate the presence of the endogenous SEI1/HDAC1/FOXC1 complex in myeloma cells, we performed immunoprecipitation procedures utilizing anti-SEI1, HDAC1, or FOXC1 antibodies on myeloma cell lysates. We checked the lysates for the presence of HDAC1, FOXC1, or SEI1. The findings revealed the presence of HDAC1, FOXC1, or SEI1 in the immunoprecipitates, which suggests that the SEI1/HDAC1/FOXC1 complex exists in myeloma cells (Figure 4B). In addition, pull-down experiments were conducted by combining lysates from HEK293T cells expressing HA-tagged SEI1, HDAC1, or FOXC1 with those expressing FOXC1 or SEI1, followed by precipitating of precipitating the complexes with an anti-HA antibody. Our results demonstrated direct interactions among SEI1, HDAC1, and FOXC1 proteins (Figure 4C). To validate these interactions, co-immunoprecipitation assays were performed in HEK293T cells co-transfected with the indicated combinations of SEI1, HDAC1, and FOXC1. Immunoprecipitation of FOXC1 co-precipitated SEI1 and HDAC1; immunoprecipitation of HDAC1 co-precipitated SEI1 and FOXC1; and immunoprecipitation of SEI1 co-precipitated HDAC1 and FOXC1 (Figure 4D). Immunofluorescence analysis also demonstrated co-localization among SEI1, HDAC1, and FOXC1 proteins (Figure 4E). These results demonstrate that SEI1 interacted with HDAC1 and FOXC1.

Previous research has revealed four conserved regions within the SEI1 protein sequence that mediate protein-protein interactions: the cyclin A-binding domain (CABD), SERTA domain (SD), PHD-bromo interaction domain (PBID), and C-terminal domain (CT).^22^ To identify which domains interact with HDAC1 or FOXC1, we engineered SEI1 mutants lacking each of the four domains (ΔCABD, ΔSD, ΔPBID, or ΔCT) (Figure 4F). Pull-down assays revealed that SEI1 binds to FOXC1 through its SD and CT domains (Figure 4G), and with HDAC1 via its SD domain (Figure 4H). To show how FOXC1 binds to the *p53* promoter, we analyzed a 1.2-kb area around the *p53* gene transcription start site. From the promoter sequence, we anticipate two FOXC1-binding sites at −566 bp and −432 bp (Figure 4I). We made two truncated versions of the *p53* promoter. Mutating both assumed sites confirmed FOXC1 binds to both sites (Figure 4J). Together, these results showed that SEI1 facilitates the interaction between HDAC1 and the FOXC1 complex to inhibit *p53* transcription.

### SEI1 strengthens the HDAC1-FOXC1 interaction, leading to a decrease in H3K27 acetylation at the *p53* promoter

**Figure 5:**
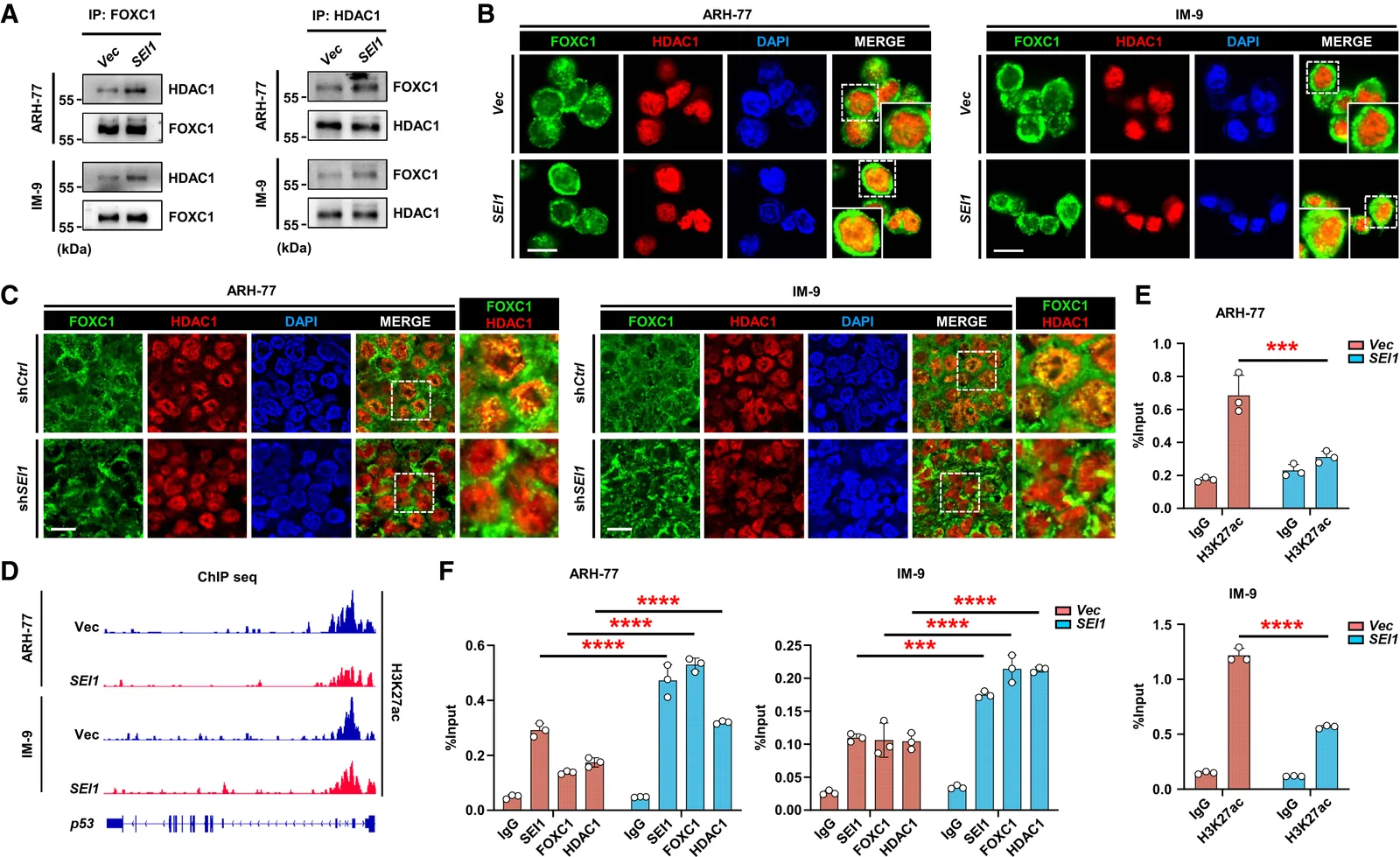
SEI1 enhances the interaction between HDAC1 and FOXC1, thereby reducing the H3K27 acetylation level at the *p53* promoter (A) Co-immunoprecipitation of FOXC1 with HDAC1 in ARH-77 or IM-9 cells (*Vec* or *SEI1*). (B) Immunofluorescent staining of ARH-77 or IM-9 cells (*Vec* and *SEI1*) with DAPI and antibody against HDAC1 and FOXC1. Scale bars, 10 μm. (C) Immunofluorescent staining of tumor tissues in Figure 2H with DAPI and antibodies against HDAC1 and FOXC1 (*n* = 3 mice/group). Scale bars, 10 μm. (D) ChIP-seq profiles show H3K27ac signal tracks at the *p53* gene loci for ARH-77 or IM-9 cells (*Vec* and *SEI1*). (E) ChIP- PCR assay showing H3K27ac enrichment on *p53* promoter of ARH-77 or IM-9 cells (*Vec* and *SEI1*). (F) ChIP-PCR assay showing SEI1, FOXC1, or HDAC1 enrichment on *p53* promoter of ARH-77 or IM-9 cells (*Vec* and *SEI1*). (A), (B), (E), and (F) are representative of three independent experiments. Data are averages ±SD. ∗∗∗*p* < 0.001, ∗∗∗∗*p* < 0.0001. (E) and (F): *p* values were determined using one way ANOVA.

Our next step was to verify the role of SEI1 within the HDAC1-FOXC1 complex. Co-immunoprecipitation assays showed that overexpression of SEI1 in myeloma cells enhanced the interaction between HDAC1 and FOXC1 (Figure 5A), whereas knockdown of SEI1 inhibited their interaction (Figure S3). Immunofluorescence analysis also demonstrated that SEI1 overexpression increased the colocalization of HDAC1 and FOXC1 in these cells (Figure 5B). Furthermore, immunofluorescence examination of subcutaneous xenograft tumors in mice confirmed that SEI1 knockdown in myeloma cells reduced HDAC1-FOXC1 colocalization (Figure 5C). Genome-wide chromatin immunoprecipitation sequencing (ChIP-seq) showed that SEI1 overexpression decreased acetylation of histone H3 at lysine 27 (H3K27ac) enrichment at the *p53* promoter (Figure 5D) but not at p53 target gene promoters (*p21*, *PUMA*, *TIGAR*) (Figures S4A–S4C), indicating that SEI1 suppresses *p53* transcription mainly via reduced H3K27ac without directly affecting p53 target genes. Notably, global H3K27ac profiling indicates that the SEI1 complex acts selectively, and further genome-wide studies are required to comprehensively delineate the transcriptional networks it governs in myeloma. Similar results were obtained using ChIP-PCR analysis (Figure 5E). Additionally, ChIP-PCR showed that SEI1 overexpression significantly increased the recruitment of SEI1, HDAC1, and FOXC1 to the *p53* promoter (Figure 5F). Moreover, ChIP-PCR analysis further demonstrated that knockdown of SEI1 in myeloma cells increased H3K27ac enrichment at the *p53* promoter (Figure S4D) and reduced the recruitment of SEI1, HDAC1, and FOXC1 to the *p53* promoter (Figure S4E). Collectively, these findings demonstrate that SEI1 strengthens the HDAC1-FOXC1 interaction, thereby reducing H3K27ac levels at the *p53* promoter, inhibiting *p53* transcription, and promoting myeloma progression.

### FOXC1 inhibitor reduces myeloma progression

**Figure 6:**
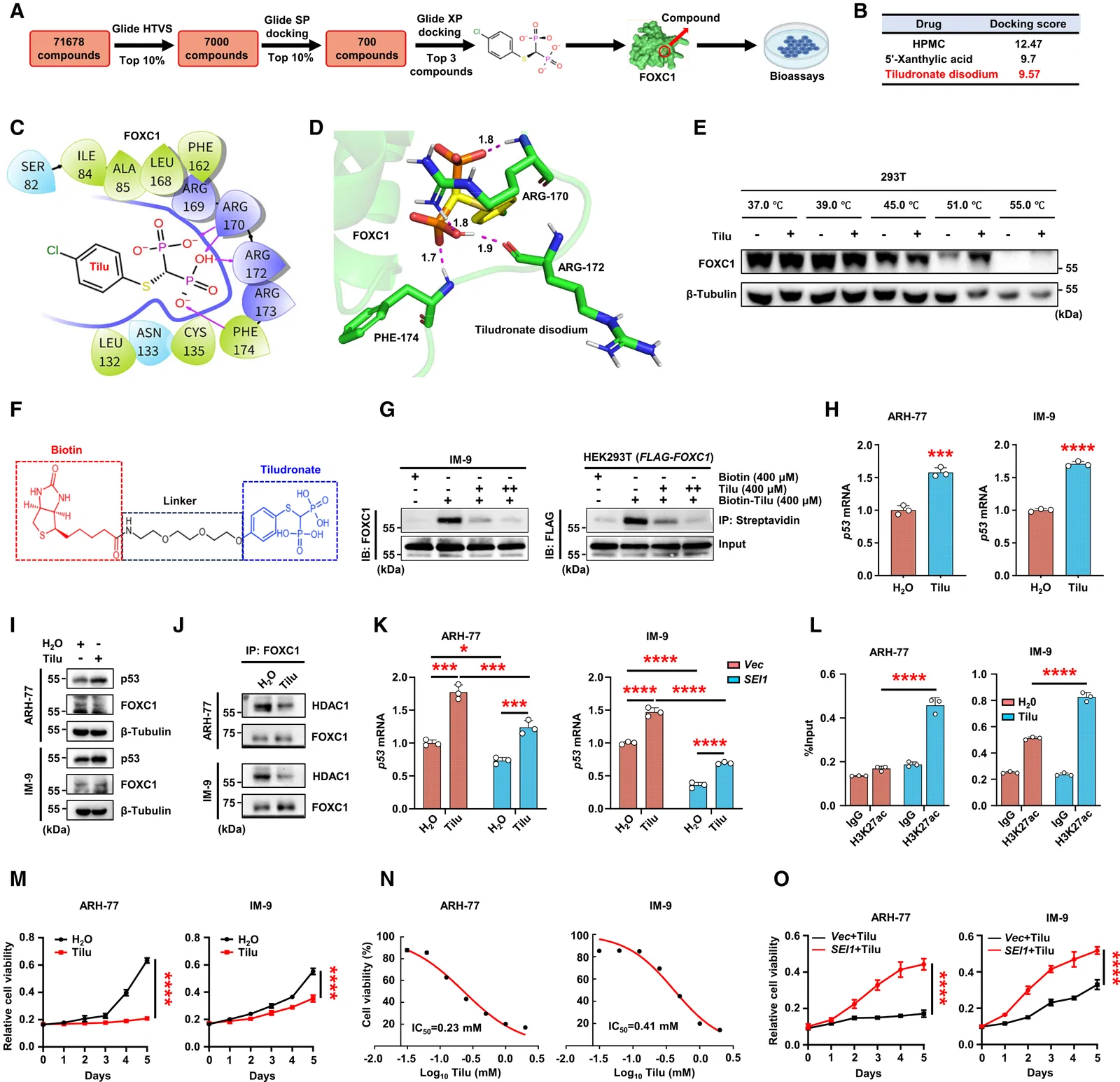
High-throughput screening and *in vitro* activity validation of FOXC1 inhibitor (A) Workflow of high-throughput virtual screening (HTVS) for FOXC1 inhibitors. (B) Docking scores of the top 3 candidates obtained from HTVS. (C and D) Chemical structure and *in silico* docking of tiludronate disodium into the active pocket of human FOXC1 protein. (E) The expression of FOXC1 protein in cellular thermal shift assay (CETSA) in HEK293T cells transfected with FOXC1 plasmid and treated with tiludronate disodium (400 μM). (F) Molecular structure of biotinylated tiludronate disodium (Biotin-Tilu). (G) Pull-down assay using lysates from IM-9 cells (left) or HEK293T cells transfected with the *FLAG-FOXC1* plasmid (right) in the presence of tiludronate disodium to examine competition between tiludronate disodium and Biotin-Tilu for FOXC1 binding. (H) Relative mRNA expression of p53 in ARH-77 or IM-9 cells treated with or without tiludronate disodium (400 μM). (I) Protein levels of p53 and FOXC1 in ARH-77 or IM-9 cells treated with or without tiludronate disodium (400 μM). (J) Co-immunoprecipitation of FOXC1 with HDAC1 in ARH-77 or IM-9 cells treated with or without tiludronate disodium (400 μM). (K) Shown are the relative expression of *p53* in ARH-77 or IM-9 cells (*Vec* or *SEI1*) treated with or without tiludronate disodium (400 μM). (L) ChIP-PCR assay showing H3K27ac enrichment on *p53* promoter of ARH-77 or IM-9 cells treated with or without tiludronate disodium (400 μM). (M) Proliferation of myeloma cells (ARH-77 or IM-9) treated with or without tiludronate disodium (400 μM) over time. (N) Antiproliferative activities of tiludronate disodium were evaluated on human myeloma cell lines ARH-77 or IM-9. (O) Proliferation of myeloma cells with increased (*SEI1*) SEI1 expression over time treated with tiludronate disodium (400 μM). Vector control (*Vec*) served as control. (E) and (G–O) are representative of three independent experiments. Data are averages ±SD. ∗*p* < 0.05, ∗∗∗*p* < 0.001, ∗∗∗∗*p* < 0.0001. (H): *p* values were determined by unpaired two-tailed *t* test; (K) and (L): *p* values were determined using one-way ANOVA; (M) and (O): *p* values were determined using two-way ANOVA.

No commercial FOXC1 inhibitors are currently available. Thus, we performed a structure-based high-throughput virtual screening of 71,678 compounds to identify potential FOXC1 inhibitors (Figure 6A). A stepwise docking approach, using standard precision (SP) followed by extra precision (XP), yielded the three top-scoring compounds for experimental validation (HPMC, 5′-Xanthylic acid tiludronate disodium) (Figure 6B). Among them, tiludronate disodium showed the strongest inhibitory effect on myeloma cells proliferation. *In silico* docking showed tiludronate disodium forms a complex with FOXC1 via four hydrogen bonds with Arg170, Phe174, and Arg158 (Figures 6C and 6D). To evaluate whether tiludronate disodium engages FOXC1 in cells using unlabeled protein and inhibitor, we applied the cellular thermal shift assay (CETSA).^23^ After treating 293T cells with 0.4 mM tiludronate disodium for 30 min, we observed significant thermal stabilization of the FOXC1 protein (Figure 6E). To further validate the interaction between tiludronate disodium and FOXC1, we conjugated biotin to the carboxyl group of tiludronate disodium (bio-Tilu) (Figure 6F) and performed a biotin pull-down assay, which confirmed the direct interaction between bio-Tilu and FOXC1; this binding was competitively inhibited by the addition of unlabeled tiludronate disodium (Figure 6G). Quantitative real-time PCR and western blot assays demonstrated that tiludronate disodium treatment elevated myeloma cells p53 expression (Figures 6H and 6I). Immunoprecipitation assays demonstrated that tiludronate disodium can significantly inhibit the interaction between FOXC1 and HDAC1 proteins (Figure 6J). Additionally, tiludronate disodium can reverse the further suppression of p53 expression caused by SEI1 over-expression (Figure 6K). Furthermore, tiludronate disodium treatment can enhance H3K27ac enrichment at the *p53* promoter region (Figure 6L).

**Figure 7:**
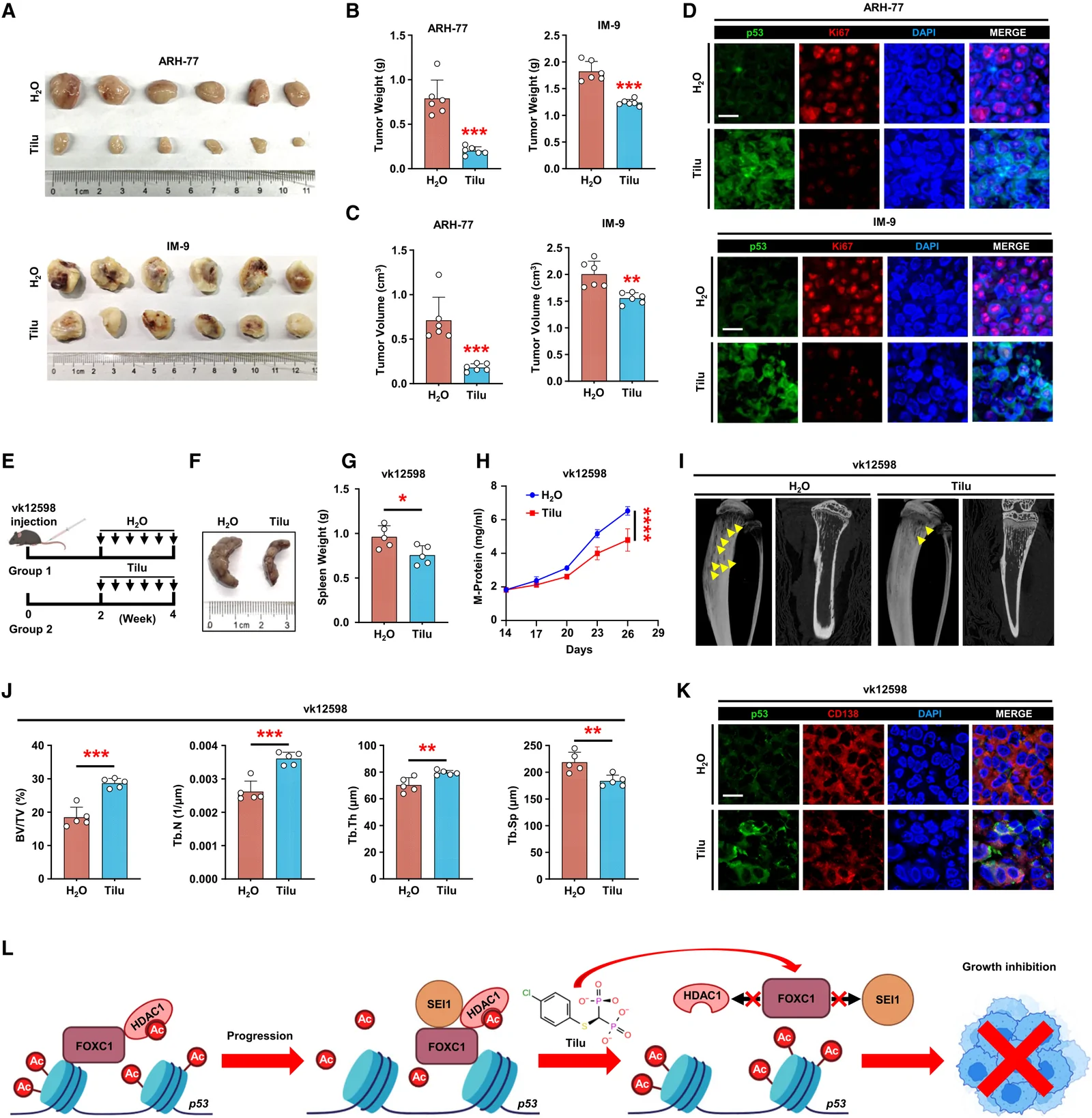
FOXC1 inhibitor tiludronate disodium reduces myeloma progression *in vivo* (A) Photographic images of tumors in NSG mice after implantation of ARH-77 or IM-9 cells (*n* = 6 mice/group), and intraperitoneal administration with or without tiludronate disodium (25 mg/kg bodyweight). (B and C) Shown are tumor weight (B) and tumor volume (C). (D) Immunofluorescent staining of Ki67 and p53 expression in tumor tissues. Scale bars, 20 μm. 6-week-old male C57BL/6J mice were intravenously injected vk12598 cells (*n* = 6 mice/group), followed by intraperitoneal administration of tiludronate disodium (25 mg/kg bodyweight) three times per week for a duration of two weeks, beginning two weeks after cell injection. (E) Shown are the experimental schematic. (F) Representative images of mouse spleens (*n* = 5 mice/group). (G and H) Shown are spleens weight (G) and time course of M protein levels (H). (I) Shown are the representative microcomputed tomography images in mouse model. (J) Percentages of BV/TV, Tb.N, Tb. Th, and Tb.Sp in mouse model treated with or without tiludronate disodium. (K) Immunofluorescent staining of F mentioned spleen tumor cells of vk12598 mice with DAPI and antibodies against CD138 and p53 (*n* = 5 mice/group). Scale bars, 20 μm. (L) Depiction of SEI1-mediated signaling pathways in myeloma cells. Data are averages ±SD. ∗∗*p* < 0.01, ∗∗∗*p* < 0.001, ∗∗∗∗*p* < 0.0001. (B), (C), (G), and (J): *p* values were determined by unpaired two-tailed *t* test; (H): *p* values were determined using two-way ANOVA.

Cell proliferation experiments demonstrated that tiludronate disodium inhibited the proliferation of myeloma cells (Figures 6M and 6N). Furthermore, SEI1 overexpression partially reversed the tiludronate disodium-induced inhibition of myeloma cell proliferation (Figure 6O). Xenograft models in NSG mice also demonstrated that tiludronate disodium inhibited the growth of ARH-77 or IM-9 cells (Figures 7A–7C). Immunofluorescence staining results also indicated that tiludronate disodium can suppress tumor proliferation and increase the expression of p53 protein (Figure 7D). We also intravenously injected vk12598 cells into C57BL/6J mice, and after 2 weeks, treated them with tiludronate disodium for two weeks (Figure 7E). The results indicated that tiludronate disodium significantly inhibited the progression of myeloma compared to control group (Figures 7F–7H). Micro-CT analysis showed that tiludronate disodium-treated myeloma-bearing mice had fewer bone lesions and lower trabecular separation (Tb.Sp), along with higher bone volume/total volume (BV/TV), trabecular number (Tb.N), and trabecular thickness (Tb. Th) in the bone chips (Figures 7I and 7J). Immunofluorescence results also confirmed that tiludronate disodium significantly increased the expression of p53 protein in myeloma cells (Figure 7K). Moreover, tiludronate disodium showed no significant cytotoxicity toward normal peripheral blood mononuclear cells (PBMCs) (Figure S5). Furthermore, PBMCs did not affect the inhibitory efficiency of tiludronate disodium (Figure S6). Overall, these findings indicate broader implications for understanding the pathophysiological mechanisms of myeloma progression and suggest potential therapeutic agents for myeloma management. In addition, cell proliferation assays indicated that tiludronate disodium significantly inhibited the proliferation of the more myeloma cell lines, lymphoma and breast cancer cell lines (Figures S7A–S7C), demonstrating its potential as a therapeutic agent in lymphoma and breast cancer treatment.

## Discussion

Improving the prognosis of myeloma requires a deeper understanding of its molecular basis. Current therapies, including radiotherapy, chemotherapy, surgery, immunotherapy, and stem cell transplantation have provided only limited survival benefits, highlighting the need for more targeted approaches. To address this, we investigated the molecular mechanisms driving myeloma progression and identified a pathogenic axis involving SEI1. Our study revealed that SEI1 is significantly overexpressed in myeloma cells, where it forms a transcriptional complex with HDAC1 and FOXC1. This complex represses *p53* transcription by reducing histone H3K27 acetylation at the *p53* promoter, leading to decreased *p53* expression and enhanced tumor proliferation. SEI1 also stabilizes the interaction between HDAC1 and FOXC1, amplifying p53 suppression and accelerating disease progression. Through high-throughput drug screening, we identified tiludronate disodium as an effective FOXC1 inhibitor. This compound disrupts the HDAC1-FOXC1 interaction, reactivates *p53* transcription, and significantly inhibits myeloma growth (Figure 7L). Collectively, these results delineate a previously unrecognized SEI1/HDAC1/FOXC1/p53 pathway in myeloma pathogenesis and establish SEI1 as a potential therapeutic target. This work advances the molecular understanding of myeloma and supports the development of more precise treatment strategies.

p53 is a tumor suppressor transcription factor that plays a crucial role in regulating the cell cycle, apoptosis, DNA repair, and other cellular processes. It helps maintain genomic stability and prevents the development of cancer.^24^ HDAC1 is overexpressed in many cancers. It promotes tumorigenesis by deacetylating histones, leading to chromatin condensation and transcriptional repression of tumor-suppressor genes.^25^ Some studies have demonstrated that in certain tumor cells, enhanced HDAC1 activity increases p53 deacetylation, rendering the p53 protein more susceptible to degradation.^26^ This ultimately weakens its regulatory function in cell-cycle control and apoptosis. Through its deacetylation action, HDAC1 can impede the binding of p53 to the promoters of its target genes, thereby reducing the transcriptional activation of downstream genes, such as p21 and PUMA.^27^ Consequently, this leads to a compromise in cell-cycle arrest and apoptosis. In myeloma, common *p53* gene alterations include loss of the short arm of chromosome 17 (17p deletion), mutations, and promoter methylation.^28^ These changes diminish p53 expression, disrupting its normal functions of cell-cycle regulation, apoptosis induction, and DNA repair.^14^ This disruption allows damaged cells to proliferate, promoting tumor growth. Previous study also showed that p53 alterations are linked to poor prognosis and drug resistance.^28^ However, our work revealed that SEI1 facilitates the HDAC1-FOXC1 interaction. By reducing H3K27 acetylation at the *p53* promoter, SEI1 inhibits *p53* transcription, thereby expanding the understanding of how HDAC1 regulates *p53* expression. Also, evidence showed that p53 mutants lack wild-type tumor-suppressor functions and acquire tumor-promoting roles.^28^ Given that 5%–8% of newly diagnosed myeloma patients have p53 mutations,^29^ whether SEI1 regulates p53 transcription via a similar mechanism in these patients remains to be studied. Additionally, current strategies to target the p53 pathway in myeloma include restoring p53 function by reactivating p53 proteins. Another approach focuses on disrupting the interaction between p53 and its negative regulators, such as mouse double minute 2 homolog (MDM2), aiming to reactivate p53 tumor-suppressor activity.^30^ Our study indicated that tiludronate disodium acts as a potential inhibitor that can reactivate the p53 pathway. These strategies aim to improve treatment outcomes and offer therapeutic options for patients with myeloma.

Tiludronate disodium is a bisphosphonate primarily used to treat Paget’s disease and postmenopausal osteoporosis by inhibiting osteoclast activity, reducing bone destruction, and increasing bone density.^31^ Our study revealed, for the first time, the therapeutic potential of tiludronate disodium in myeloma. Specifically, tiludronate disodium targeted FOXC1, disrupted the FOXC1-HDAC1 interaction, and activated p53, thereby inhibiting myeloma cell proliferation and reducing osteolytic bone lesions. These findings suggest tiludronate disodium may be a promising agent for myeloma and its complications, expanding its clinical applications. Whether tiludronate disodium combined with existing therapies (e.g., chemotherapy, immunotherapy) can enhance efficacy is a promising avenue for future myeloma research. Furthermore, FOXC1 is involved in cancer progression, including migration, invasion, and cancer stem cell regulation, and also plays key roles in physiological processes, such as stem cell niche maintenance, epithelial-mesenchymal transition, and angiogenesis.^21^ Given that FOXC1 functions as an oncogene, tiludronate disodium holds therapeutic potential in cancer treatment, warranting further investigation in future studies.

### Limitations of the study

Despite the insights provided by this study, several limitations should be acknowledged. Our results demonstrated that SEI1 expression increases during myeloma progression. It interacts with FOXC1 and HDAC1 to suppress *p53* transcription by reducing H3K27 acetylation at its promoter, thereby promoting tumor progression. However, prior work has shown that chemotherapy can upregulate SEI1 through the DNA damage/cGAS/STING/IRF7 pathway. In that context, SEI1 recruits CBP/p300 and the PAF1 complex to enhance H3K27 acetylation at the *PD-L1* promoter and increase PD-L1 expression.^19^ Thus, SEI1 may interact with both histone deacetylase and acetyltransferase complexes in myeloma, but the mechanisms governing these opposing functions and their potential co-occurrence remain unclear and require further investigation.

## Resource availability

### Lead contact

Further information and requests for resources should be directed to and will be fulfilled by the lead contact, Huan Liu (huanliu@xmu.edu.cn).

### Materials availability

This study did not generate new materials.

### Data and code availability

• **Data**: The RNA-seq data generated in this study are available at BioProject (PRJNA1272163). ChIP-seq data generated in this study is available at BioProject (PRJNA1222270).
• **Code**: This study did not generate a new code.
• **Other**: Any additional information required to reanalyze the data reported in this paper is available from the lead contact upon request.

## Acknowledgments

We thank the Core Facility of Biomedical Sciences of 10.13039/501100008865Xiamen University. This research was supported by the 10.13039/501100003392Natural Science Foundation of Fujian Province (2022D015, 2024R1036 to H.L.), 10.13039/501100021171Guangdong Basic and Applied Basic Research Foundation (2024A1515010076 to H.L.), 10.13039/501100001809National Natural Science Foundation of China (82570252 to H.L.), The Science and Technology Innovation Leading Talent Support Program of Henan Province (254000510004 to C.W.), and Key Scientific Research Funding Program for Higher Education Institutions of Henan Province (24A320060 to C.W.).

## Author contributions

H.L. and C.W. designed all the experiments and wrote the manuscript. R.C., R.L., Z.H.F., D.Y.Y., Z.L., Yuan Li (author 6), Yuan Li (author 7), S.R.L., Q.L., and Y.Q.C. performed the experiments and statistical analysis. Z.H.F. and C.W. provided the patient samples. All authors have reviewed the final manuscript.

## Declaration of interests

The authors declare no conflicts of interest.

## Declaration of generative AI and AI-assisted technologies in the writing process

During the preparation of this work, the author(s) used DeepSeek-an AI language model-to check grammar and language. After using this tool, the author(s) reviewed and edited the content as needed and take full responsibility for the content of the publication.

## STAR★Methods

### Key resources table

**Table:** 

REAGENT or RESOURCE	SOURCE	IDENTIFIER
**Antibodies**		

β-Tubulin	Cell Signaling Technology	Cat# 2146
p53	Cell Signaling Technology	Cat# 2527
HDAC1	Cell Signaling Technology	Cat# 34589; RRID: AB_2756821
FOXC1	Cell Signaling Technology	Cat# 8758; RRID: AB_2797657
Flag	Cell Signaling Technology	Cat# 14793; RRID: AB_2572291
HA	Cell Signaling Technology	Cat# 3724; RRID: AB_1549585
SEI1	Santa Cruz Biotechnology	Cat# sc-517080
H3K27ac	Cell Signaling Technology	Cat# 8173; RRID: AB_10949503
HDAC1	Proteintech	Cat# 66085-1-Ig; RRID: AB_11232033
FITC-conjugated Goat anti-Mouse IgG	Abclonal	Cat# AS001
Cy3-conjugated Goat anti-Rabbit IgG	Abclonal	Cat# AS007
CD138	R&D Systems	Cat# AF2780
Ki67	Proteintech	Cat# 66555-6-Ig; RRID: AB_3743304
Cy5-conjugated Mouse Anti-Goat IgG	Solarbio	Cat# K0038M-Cy5
Normal rabbit IgG	Santa Cruz Biotechnology	Cat # sc-2027; RRID: AB_737197
Normal mouse IgG	Santa Cruz Biotechnology	Cat # sc-2025; RRID: AB_737182
Normal goat IgG	Santa Cruz Biotechnology	Cat # sc-2028; RRID: AB_737167
Protein G agarose	Thermo Fisher Scientific	Cat # 22852
anti-HA agarose	Thermo Fisher Scientific	Cat # 26182; RRID: AB_2532162

**Biological samples**		

Bone marrow derived myeloma cells	Patient bone marrow aspirates	In-house

**Chemicals, peptides, and recombinant proteins**		

DAPI	Sigma Aldrich	Cat# D9542
Tiludronate disodium	TargetMol	Cat# T13159
Biotin	MedChemExpress LLC	Cat# HY-B0511
Streptavidin Magnetic Beads	MedChemExpress LLC	Cat# HY-K0208
Propidium iodide solution	Thermo Fisher Scientific	Cat# P3566

**Critical commercial assays**		

IgG2b Mouse Uncoated ELISA Kit	Thermo Fisher Scientific	Cat# 88-50430-88
SimpleChIP Plus Enzymatic Chromatin IP Kit	Cell Signaling Technology	Cat# 9004
SignalFire Plus ECL Reagent	Cell Signaling Technology	Cat# 12630
EdU assay kit	RiboBio	Cat# 10310
Dead Cell Apoptosis Kit with Annexin V FITC & Propidium Iodide for Flow Cytometry	Thermo Fisher Scientific	Cat# V13242

**Deposited data**		

RNA-seq	This manuscript	BioProject (PRJNA1272163)
ChIP-seq	This manuscript	BioProject (PRJNA1222270)

**Experimental models: Cell lines**		

Human myeloma cell lines: ARH-77, IM-9, ARP-1, and LP-1	Gift from Dr. Zhiqiang Liu	N/A
Human lymphoma cell lines: JeKo-1, and Jurkat	Gift from Dr. Zhiqiang Liu	N/A
Mouse myeloma cell line: vk12598	Gift from Mayo Clinic	N/A
Human breast cancer cell line: MCF-7	ATCC	Cat# HTB-22; RRID: CVCL_0031
Human breast cancer cell line: MDA-MB-231	ATCC	Cat# HTB-26; RRID: CVCL_0062
HEK293T	ATCC	Cat# CRL-3216; RRID: CVCL_0063

**Experimental models: Organisms/strains**		

Mouse: C57BL/6J	Shanghai Model Organisms Center	Cat# SM-001
Mouse: NSG(NOD.Cg-*Prkdc*^*scid*^*Il2rg*^*em1Smoc*^)	Shanghai Model Organisms Center	Cat# NM-NSG-001

**Oligonucleotides**		

See Supplementary Table for primers	This manuscript	N/A

**Recombinant DNA**		

pLVx-AcGFP-N1 vector	NovoPro	Cat# V012707
pLKO.1-puro	Addgene	Cat# 8453
pLVx-*SEI1*	This manuscript	N/A
pLVx-*HA-SEI1*	This manuscript	N/A
pLVx-*HA-HDAC1*	This manuscript	N/A
pLVx-*FLAG-FOXC1*	This manuscript	N/A
pLVx-*MYC-SEI1*	This manuscript	N/A
pLKO.1-sh*SEI1*	This manuscript	N/A
pGL2 Luciferase Reporter Vector	Promega	Cat# TM003
pGL2-*p53-Full*	This manuscript	N/A
pGL2-*p53-Mut1*	This manuscript	N/A
pGL2-*p53-Mut2*	This manuscript	N/A

**Software and algorithms**		

Flowjo software version 10	FlowJo LLC	https://www.flowjo.com/
GraphPad PRISM 10.0	GraphPad Software	https://www.graphpad.com/
ImageJ	NIH	https://imagej.nih.gov/ij/

### Experimental model and study participant details

#### Animal models

Female NSG mice (6 weeks old) and female C57BL/6J mice (6 weeks old) were obtained from Shanghai Model Organisms Center. Mice were housed five per cage under specific pathogen-free (SPF) conditions in a temperature- and humidity-controlled facility (20°C–25°C, 45%–64% humidity), with a 12-h light/dark cycle and *ad libitum* access to food and water. All animal experiments were approved by the Institutional Animal Care and Use Committee of Xiamen University (approval number: XMULAC20230119). Animals were randomly assigned to experimental groups, and investigators were blinded to group allocation during tumor measurements. Only female mice were used in this study. Therefore, potential sex-related differences could not be evaluated. Mouse models used in this study were described in the method details.

#### Patient samples

Bone marrow aspirates were obtained from two distinct cohorts. The myeloma patient cohort comprised 20 newly diagnosed myeloma patients (median age 67.4 years, range 47–85; 11 male and 9 female) at the First Affiliated Hospital of Xiamen University. Samples were consecutively enrolled based on the following inclusion criteria: confirmed diagnosis of active multiple myeloma, CD138^+^ myeloma cell infiltration ≥10% in bone marrow, and no prior anti-myeloma therapy. Exclusion criteria included active infection, other concurrent malignancies, or refusal to provide informed consent. The healthy control cohort comprised 20 age- and sex-matched healthy donors (median age 66.7 years, range 42–80; 10 male and 10 female) who underwent bone marrow examination for non-malignant workup and were confirmed to have no hematological or malignant disorders. Control samples were randomly selected from the institutional biobank to match the patient cohort in age and sex distribution. Primary myeloma cells and normal plasma cells were isolated from the respective bone marrow aspirates using anti-CD138 magnetic microbeads (Miltenyi Biotec). No sample size calculation was performed prior to collection; the sample size was determined by the availability of eligible specimens during the study period. Written informed consent was obtained from all participants prior to sample collection. The characteristics of the patients among different groups are compatible, where there was no significant difference between our samples from male and female patients. The study protocol was reviewed and approved by the Ethics Committee of Xiamen University and was conducted in accordance with the principles of the World Medical Association Declaration of Helsinki (approval number: 2025193).

#### Cell cultures

Myeloma cell lines (ARH-77 [female], IM-9 [female], ARP-1 [unknown gender], and LP-1 [female]) and lymphoma cell lines (JeKo-1 [female] and Jurkat [male]) were obtained from Dr. Zhiqiang Liu’s laboratory (Shandong First Medical University & Shandong Academy of Medical Sciences). The murine myeloma cell line vk∗MYC (vk12598) (unknown gender) was kindly provided by the Mayo Clinic. Human breast cancer cell lines (MCF-7 [female] and MDA-MB-231 [female]) and HEK293T [female] cells were purchased from the American Type Culture Collection (ATCC). All established cell lines were maintained in RPMI 1640 (myeloma and lymphoma lines) or Dulbecco’s Modified Eagle Medium (DMEM) (MCF-7, MDA-MB-231, HEK293T), supplemented with 10% fetal bovine serum (FBS) at 37°C in a humidified 5% CO_2_ incubator. All cell lines used in this study were authenticated by short tandem repeat (STR) profiling (Biowing Biotech, Shanghai, China) within the last 12 months, and their STR profiles matched the corresponding reference profiles in the ATCC or DSMZ databases. All cell lines were routinely tested and confirmed to be free of mycoplasma contamination using the Universal Mycoplasma Detection Kit (ATCC, Manassas, VA, USA).

### Method details

#### Plasmid construction, antibodies, and reagents

Expression plasmids encoding human *SEI1*, HA-tagged *SEI1*, HA-tagged *HDAC1*, Flag-tagged *FOXC1*, and Myc-tagged *SEI1* were constructed by cloning the respective sequences into the pLVx-AcGFP-N1 vector (NovoPro, #V012707). Short hairpin RNAs (shRNAs) targeting SEI1 and a non-targeting control shRNA were acquired from Sigma-Aldrich and subsequently cloned into the pLKO.1 lentiviral vector. Primers used for generating SEI1 amino acid deletion fragments are listed in Table S3. Unless specified otherwise, all chemical reagents were purchased from Sigma-Aldrich, and all antibodies for western blot analysis were obtained from Cell Signaling Technology.

#### Stable cell line construction

For transient transfection, HEK293T cells were transfected using polyethyleneimine (PEI) in OPTI-MEM medium (Life Technologies), with a DNA-to-PEI ratio of 1:4. Lentiviral particles were produced by co-transfecting HEK293T cells with 4 μg pMD2.G (#12259, Addgene), 6 μg psPAX2 (#12260, Addgene), and 8 μg of the respective lentiviral expression vector (e.g., pLVx-AcGFP-N1-SEI1 or pLKO.1-based shRNA constructs). The viral supernatant was collected 48 h post-transfection and concentrated 100-fold using Polyethylene glycol 8000 (Sigma-Aldrich). Myeloma cells were infected via spinfection at 800 × g for 30 min at 37°C in the presence of concentrated virus and polybrane. The medium was replaced after 12 h, and cells were cultured for an additional 48 h prior to experimental use. Stable cell lines were selected and maintained using puromycin (2 μg/mL; #540222, Sigma-Aldrich).

#### Western blot analysis

Cells were harvested and lysed with 1 × lysis buffer (Cell Signaling Technology). Protein lysates were separated by SDS-PAGE and subsequently transferred onto nitrocellulose membranes. Immunoblotting was performed using the following primary antibodies: β-Tubulin (#2146), p53 (#2527), HDAC1 (#34589), FOXC1 (#8758), Flag (#14793), and HA (#3724) (all from Cell Signaling Technology). An anti-SEI1 antibody (sc-517080) was purchased from Santa Cruz Biotechnology.

#### Quantitative real-time PCR of mRNAs

Total RNA was isolated using the RNeasy extraction kit (QIAGEN). One microgram of purified RNA was reverse-transcribed with the SuperScript II RT-PCR kit (Invitrogen). Quantitative real-time PCR was performed using SYBR Green Master Mix (Life Technologies) on a QuantStudio 3 Real-Time PCR System (Life Technologies). The amplification protocol consisted of an initial denaturation at 95°C for 10 min, followed by 40 cycles of 95°C for 15 s and 60°C for 1 min. *GAPDH* served as the endogenous reference gene for normalization. Primer sequences are listed in Table S4.

#### Cell viability and ELISA analysis

Cell viability was assessed using the Cell Counting Kit-8 (Dojindo). Cells were seeded in triplicate at a density of 10,000 cells per well and analyzed according to the manufacturer’s protocol. Cell proliferation was evaluated with the EdU assay kit (RiboBio), following the supplied instructions. ELISA kits were obtained from Thermo Fisher Scientific and Immunodiagnostic Systems.

#### Immunoprecipitation and pull-down assays

For immunoprecipitation, cell lysates were prepared and incubated on ice for 15 min. Total protein lysates were immunoprecipitated with agarose bead-conjugated antibodies overnight at 4°C. Beads were washed six times and resuspended in 30 μL of 1 × SDS buffer. Samples were boiled for 5 min and subjected to SDS-PAGE, alongside a 5% input control, followed by transfer to a PVDF membrane for western blot analysis. IgG and total cell lysate were used as negative and positive controls, respectively. For pull-down assays, HEK293T cells were transfected with plasmids expressing Flag- or Myc-tagged proteins. Cell lysates containing these tagged proteins were incubated with anti-Flag or anti-Myc beads, and subsequently mixed with lysates from cells expressing full-length, truncated *SEI1*, or HA-*HDAC1*. Immunoprecipitated complexes and whole cell lysates were analyzed by western blotting. Untransfected cells or cells transfected with control plasmids, along with whole lysates, served as negative controls.

#### Chromatin-immunoprecipitation (ChIP), and ChIP-sequencing (ChIP-seq)

Cells were fixed with 4% formaldehyde and chromatin was fragmented by sonication. Chromatin samples were immunoprecipitated overnight at 4°C using antibodies against HDAC1 (#34589), FOXC1 (#8758), H3K27ac (#8173) (all from Cell Signaling Technology), SEI1 (#sc-517080, Santa Cruz), or control IgG. Following cross-link reversal, DNA was purified from immunoprecipitates and total chromatin inputs and analyzed by PCR using primers listed in Table S5.

For sequencing, libraries were prepared from 20 ng of ChIP DNA (anti-H3K27ac or IgG control) using an Illumina kit (#IP-102-1001). Libraries were sequenced on a HiSeq 2000 platform (6 pM, 50 cycles). Raw reads were quality-checked with FastQC (v0.11.8) and trimmed with Cutadapt (v2.0). High-quality reads were aligned to the hg38 genome using Bowtie2 (v2.2.6). Duplicate reads were removed with SAMtools (v1.9). Peak calling was performed with MACS2 (v2.1.1) according to the ENCODE histone ChIP-seq pipeline. Genomic annotation of peaks was conducted using HOMER (v4.10) with the annotatePeaks.pl script.

#### Immunofluorescent staining

Myeloma cells were fixed with 4% formaldehyde and permeabilized with 0.3% Triton X-100. After blocking in 2% goat serum, cells were incubated overnight at 4°C with primary antibodies against SEI1 (#sc-517080, Santa Cruz), HDAC1 (#66085-1-Ig, Proteintech), HDAC1 (#34589, Cell Signaling Technology), or FOXC1 (#8758, Cell Signaling Technology). Cells were then incubated with FITC-conjugated Goat anti-Mouse IgG (#AS001, Abclonal) or Cy3-conjugated Goat anti-Rabbit IgG (#AS007, Abclonal) for 60 min at room temperature. Nuclei were counterstained with DAPI.

Formalin-fixed, paraffin-embedded sections from patient bone marrow biopsies or mouse models were deparaffinized and stained. Sections were incubated overnight at 4°C with antibodies against CD138 (#AF2780, R&D Systems; 1:100), SEI1 (#sc-517080, Santa Cruz), p53 (#2527, Cell Signaling Technology), Ki67 (#66555-6-Ig, Proteintech), HDAC1 (#66085-1-Ig, Proteintech), or FOXC1 (#8758, Cell Signaling Technology). Subsequently, samples were incubated with FITC-conjugated Goat anti-Mouse IgG (#AS001, Abclonal), Cy3-conjugated Goat anti-Rabbit IgG (#AS007, Abclonal), or Cy5-conjugated Mouse Anti-Goat IgG (#K0038M-Cy5, Solarbio) for 60 min at room temperature. Nuclei were counterstained with DAPI. Fluorescence images were acquired using an IX71 confocal microscope (Olympus). Quantitative analysis of immunofluorescence staining is provided in Figure S8.

#### Whole-transcriptome sequencing

Total RNA was extracted from ARH-77 or IM-9 cells (Vector or SEI1-overexpressing) and purified using VAHTSTM DNA Clean Beads (Vazyme). Sequencing libraries were constructed with the VAHTS Universal V6 RNA-seq Library Prep Kit (Vazyme). The pooled libraries were quantified with the Qubit dsDNA HS Assay Kit (Thermo Fisher Scientific) and their size distribution was assessed using a Fragment Analyzer (Advanced Analytical). Libraries were then sequenced on the DNBSEQ-T7 platform (MGI Tech).

#### Mass spectrometry

HA-SEI1-overexpressing IM-9 cells and vector-transfected control cells were lysed and incubated on ice for 15 min. Total protein lysates were immunoprecipitated overnight at 4°C using agarose-conjugated HA antibody. After six washes, eluted proteins were analyzed by nano-liquid chromatography-tandem mass spectrometry (nano-LC/MS/MS) using a Thermo Fisher Scientific system coupled to an 1100 HPLC (Agilent Technologies). MS/MS spectra were searched against the NCBI database using SEQUEST in BioWorks Browser (v3.3.1, Thermo Fisher Scientific). The mass spectrometry proteomics data have been deposited to the ProteomeXchange Consortium via the PRIDE repository with the dataset identifier PXD052783.

#### Luciferase assay *in vitro*

Full-length p53 (*p53-Full*) and its mutant constructs (*p53-Mut1*, *p53-Mut2*) were cloned into the pGL2 vector. Transcriptional activity was measured in HEK293T cells using the Dual-Luciferase Reporter Assay System (Promega) according to the manufacturer’s instructions. Primers used for subcloning are provided in Table S6.

#### High-throughput virtual screening (HTVS)

Structure-based high-throughput virtual screening (HTVS) was performed using Schrödinger Maestro 11.4. The three-dimensional structure of human FOXC1 (AlphaFold ID: AF-Q12948-F1) was retrieved from the AlphaFold database. The MCE Bioactive Compounds Library Plus (71,678 compounds) was energy-minimized with Schrödinger’s LigPrep module. Molecular docking was conducted through a multi-tiered workflow: primary HTVS (top 10% retained), Standard Precision (SP) docking of HTVS hits (top 10% retained), and final refinement with Extra Precision (XP) docking. The three compounds with the highest absolute docking scores (reflecting stronger predicted binding affinity) were selected for experimental validation. Tiludronate disodium (#T13159, TargetMol, USA) was obtained commercially. Three-dimensional binding poses were visualized in PyMol.

#### *In vivo* mouse experiments

To evaluate the effect of SEI1 on tumor growth, NSG mice (6-week-old females) were randomly and blindly assigned to groups (*n* = 3 per group) and subcutaneously injected with ARH-77 or IM-9 myeloma cells (1 × 10^6^ cells/mouse) expressing either control shRNA (sh*Ctrl*) or SEI1-targeting shRNA (sh*SEI1*). Tumor diameters were measured every other day. Mice were sacrificed 3 weeks post-injection, and tumors were excised, weighed, photographed, fixed in 10% neutral-buffered formalin, sectioned, and stained for Ki67 (Proteintech) using standard protocols.

Myeloma cells (ARH-77 or IM-9, 1 × 10^6^ cells/mouse) were subcutaneously injected into NSG mice (6-week-old females). Starting one week after inoculation, mice were administered tiludronate disodium (25 mg/kg body weight) or vehicle intraperitoneally three times per week for 2 weeks (*n* = 6 per group). Tumor growth was monitored every other day. After 3 weeks, mice were euthanized and tumors were collected for weighing, photography, and immunohistochemical analysis of Ki67 and p53 expression. No animals were excluded from analysis.

In C57BL/6J mice (6-week-old females), animals were randomly and blindly grouped (*n* = 5 per group) and intravenously injected with vk12598 cells. Starting 2 weeks after injection, tiludronate disodium (25 mg/kg body weight) or PBS was administered intraperitoneally three times weekly for 2 weeks. Myeloma progression was assessed by M protein detection 4 weeks post-injection. Spleens were harvested for immunofluorescence analysis. No animals were excluded from analysis.

#### Flow cytometry analysis

Apoptosis was assessed using an Annexin-V assay as previously described.^32^ Briefly, myeloma cells (1 × 10^5^ cells/mL) were stained with fluorescein isothiocyanate-conjugated annexin V and propidium iodide. For cell cycle analysis, cells were fixed with 70% ice-cold ethanol and then stained with 50 μg/mL propidium iodide supplemented with 20 μg/mL RNase. All samples were analyzed on a BD LSRFortessa flow cytometer, and the data were processed using FlowJo software.

### Quantification and statistical analysis

#### Statistical analysis

Sample sizes, replication schemes, and interim endpoint determinations were established based on previous experimental studies,^33^^,^^34^ ensuring a statistical power of 80% and a Type I error rate (α) of 0.05. Data are shown as mean ± SD. All statistical analyses were performed using GraphPad Prism (Version 9.0). Differences between two groups were evaluated using two-tailed, unpaired Student’s *t*-tests. Comparisons among multiple groups were conducted by one-way ANOVA followed by Tukey’s post hoc test or two-way ANOVA with Bonferroni’s post hoc test. Pearson’s correlation analysis was used to examine the correlation between two variables and Kaplan-Meier analysis was used in survival analysis. Homogeneity of variance was confirmed between groups under comparison. A *p* value of less than 0.05 was considered statistically significant. All statistical details of experiments can be found in the figure legends.

## Supplemental information

Document S1. Figures S1–S8 and Tables S2–S6

Table S1. Identification of SEI1-binding proteins by mass spectrometry, related to Figure 4
