# Supplementary material for: SEI1 complex silences p53 to drive myeloma progression
Source: iScience. 2026 Aug 24;29(9):117300. doi: 10.1016/j.isci.2026.117300 (PMC13542490; doi:10.1016/j.isci.2026.117300)
Supplement: Supplementary file 1 [file mmc1.pdf]

iScience, Volume *29*

Supplemental information

SEI1 complex silences p53

to drive myeloma progression

Rui Chen (陈睿), Rui Liu (刘蕊), Zhihong Fang (方志鸿), Daoyan Yang (杨道焱), Zou Li (李邹), Yuan Li (李圆), Yuan Li (李源), Shurong Liu (刘淑溶), Qi Liu (刘琦), Yanqi Chao (钞艳岐), Chong Wang (王冲), and Huan Liu (刘欢)

Supplementary Figures and Figure Legends

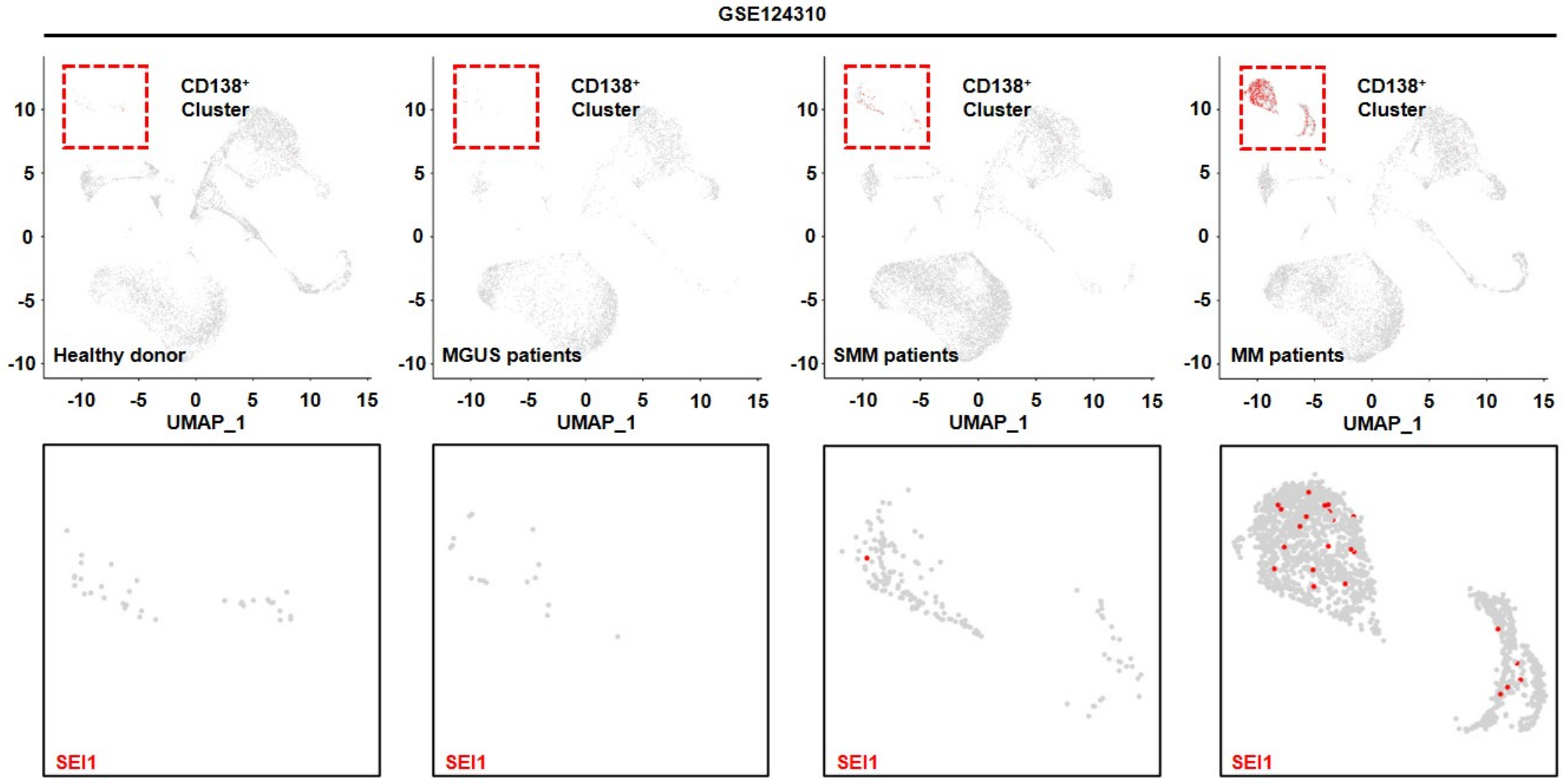

Figure S1. SEI1 expression is upregulated in myeloma. Related to Figure 1.

Representative UMAP plot of the expression of *CD138* and *SEI1* in a public scRNA-seq data (GSE124310) from a cohort of 22 patients with varying stages of myeloma progression and 9 healthy donors.

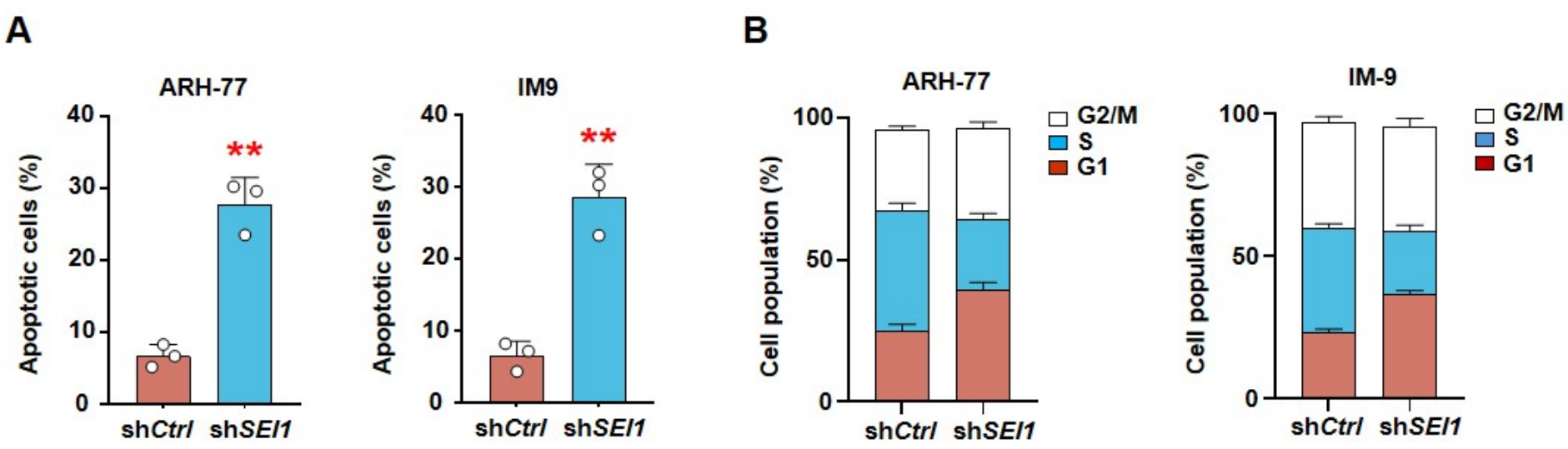

Figure S2. SEI1 knockdown increased myeloma cell apoptosis and cell cycle arrest. Related to Figure 2.

(**A**) Shown are the percentage of apoptotic myeloma cells (ARH-77 or IM9) with reduced (sh*SEI1*) SEI1 expression. Nontargeted shRNA (sh*Ctrl*) served as control. (**B**) Shown are the percentages of cell populations in G2/M, S, or G1 phase of myeloma cell cycle (sh*SEI1 or* sh*Ctrl*). All data are representative of three independent experiments. Data are averages ± SD. $^{**}P < 0.01$. P values were determined by unpaired two-tailed t test.

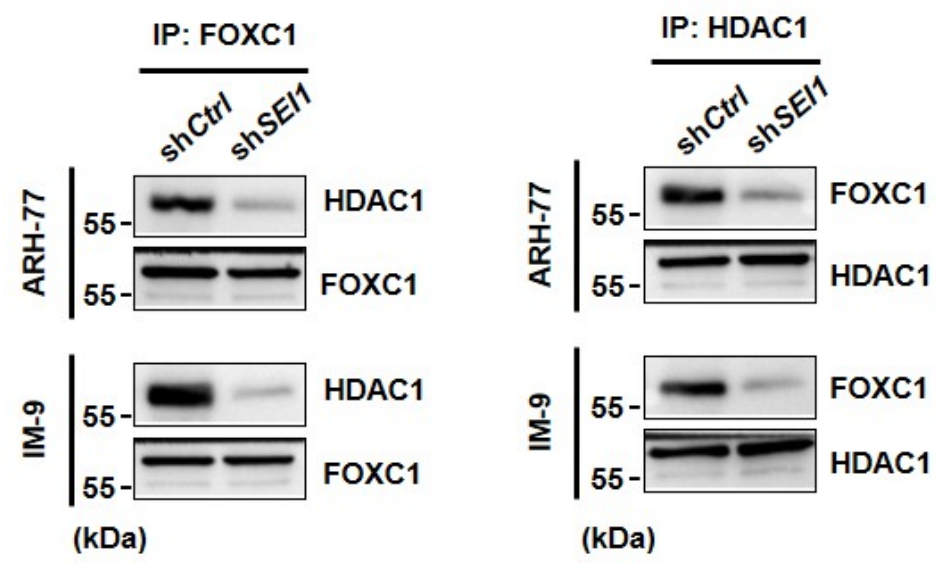

Figure S3. SEI1 enhances the interaction between HDAC1 and FOXC1. Related to Figure 5.

Co-immunoprecipitation of FOXC1 with HDAC1 in ARH-77 or IM-9 cells (sh*Ctrl* or sh*SEI1*). All data are representative of three independent experiments.

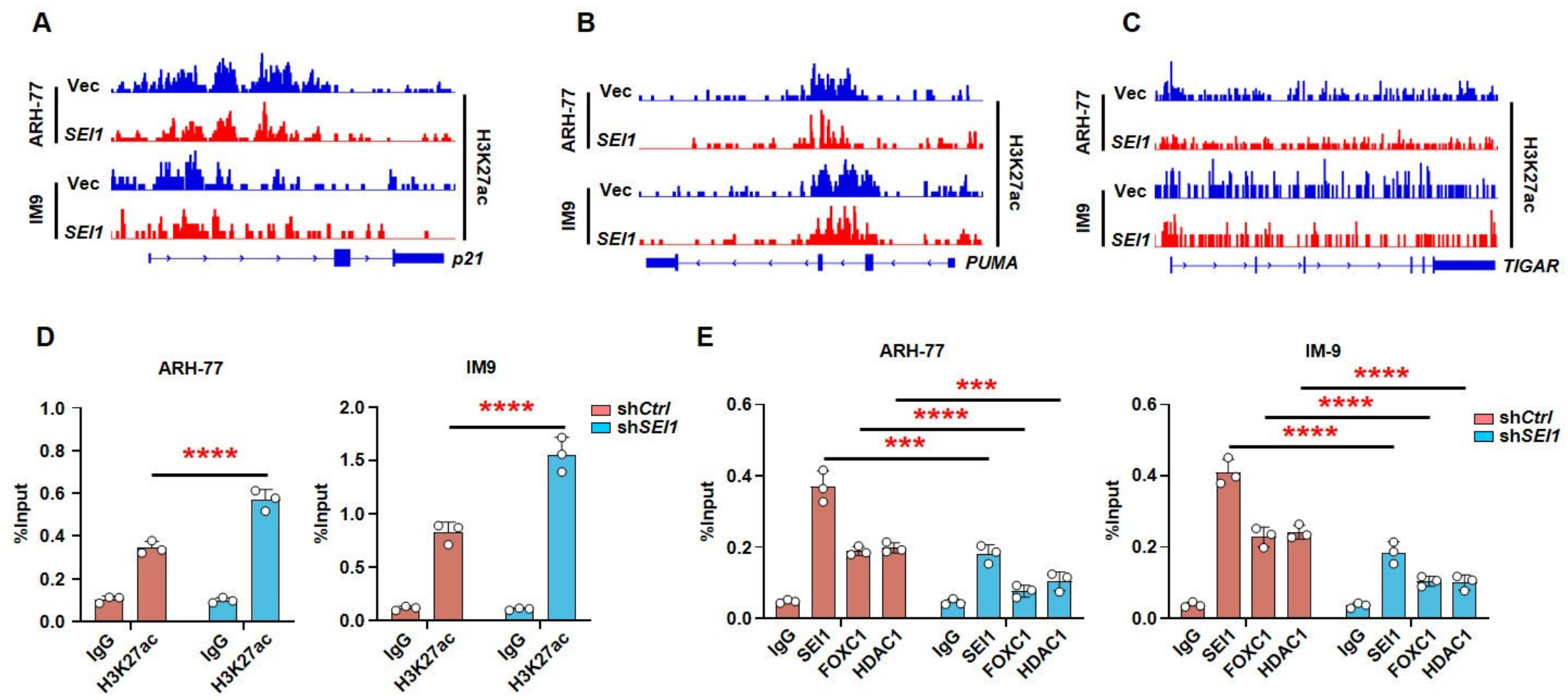

Figure S4. SEI1 reduces the H3K27 acetylation level at the *p53* promoter. Related to Figure 5.

(**A**-**C**) ChIP-seq profiles showing H3K27ac signal tracks at the *p21*, *PUMA*, or *TIGAR* gene loci for ARH-77 or IM-9 cells (*Vec* and *SEI1*). (**D**) ChIP-PCR assay showing H3K27ac enrichment on *p53* promoter of ARH-77 or IM-9 cells (sh*Ctrl* or sh*SEI1*). (**E**) ChIP PCR assay showing SEI1, FOXC1 or HDAC1 enrichment on *p53* promoter of ARH-77 or IM-9 cells (sh*Ctrl* or sh*SEI1*). **D**, and **E** are representative of three independent experiments. Data are averages ± SD. ****P* < 0.001, *****P* < 0.0001. **D**, **E**: *P* values were determined using one way ANOVA.

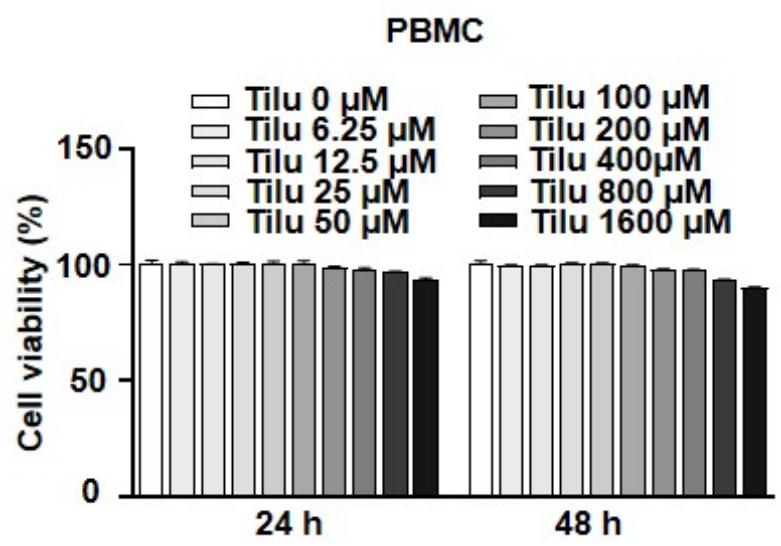

Figure S5. Tiludronate disodium shows no significant cytotoxicity to normal PBMC cells. Related to Figure 6.

Proliferation of PBMC cells treated with or without tiludronate disodium (6.25, 12.5, 25, 50, 100, 200, 400, 800 or 1600 μM) over time. PBS treated group served as control (n = 3 biological replicates). Data are averages ± SD.

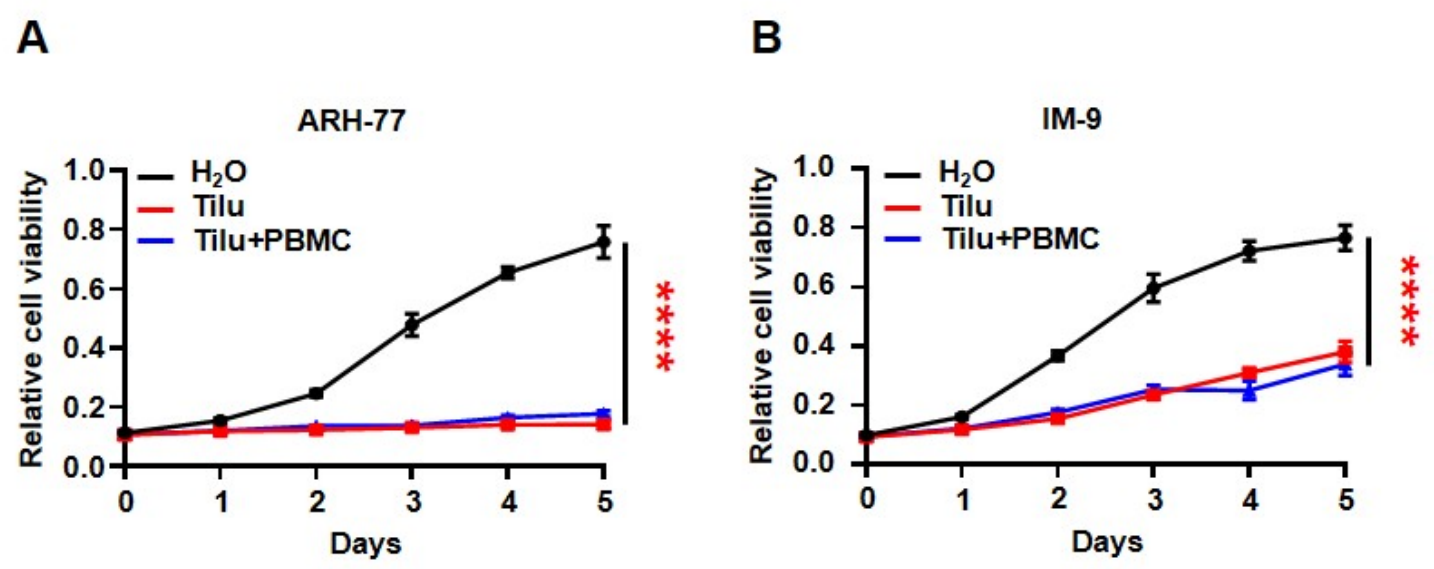

Figure S6. PBMCs do not affect the inhibitory efficiency of tiludronate disodium. Related to Figure 6.

(**A**, **B**) Proliferation of myeloma cells (ARH-77 or IM-9) treated with or without tiludronate disodium (400 µM), and cocultured with or without PBMCs over time. All data are representative of three independent experiments. Data are averages ± SD. ****$P< 0.0001$. P values were determined using two-way ANOVA.

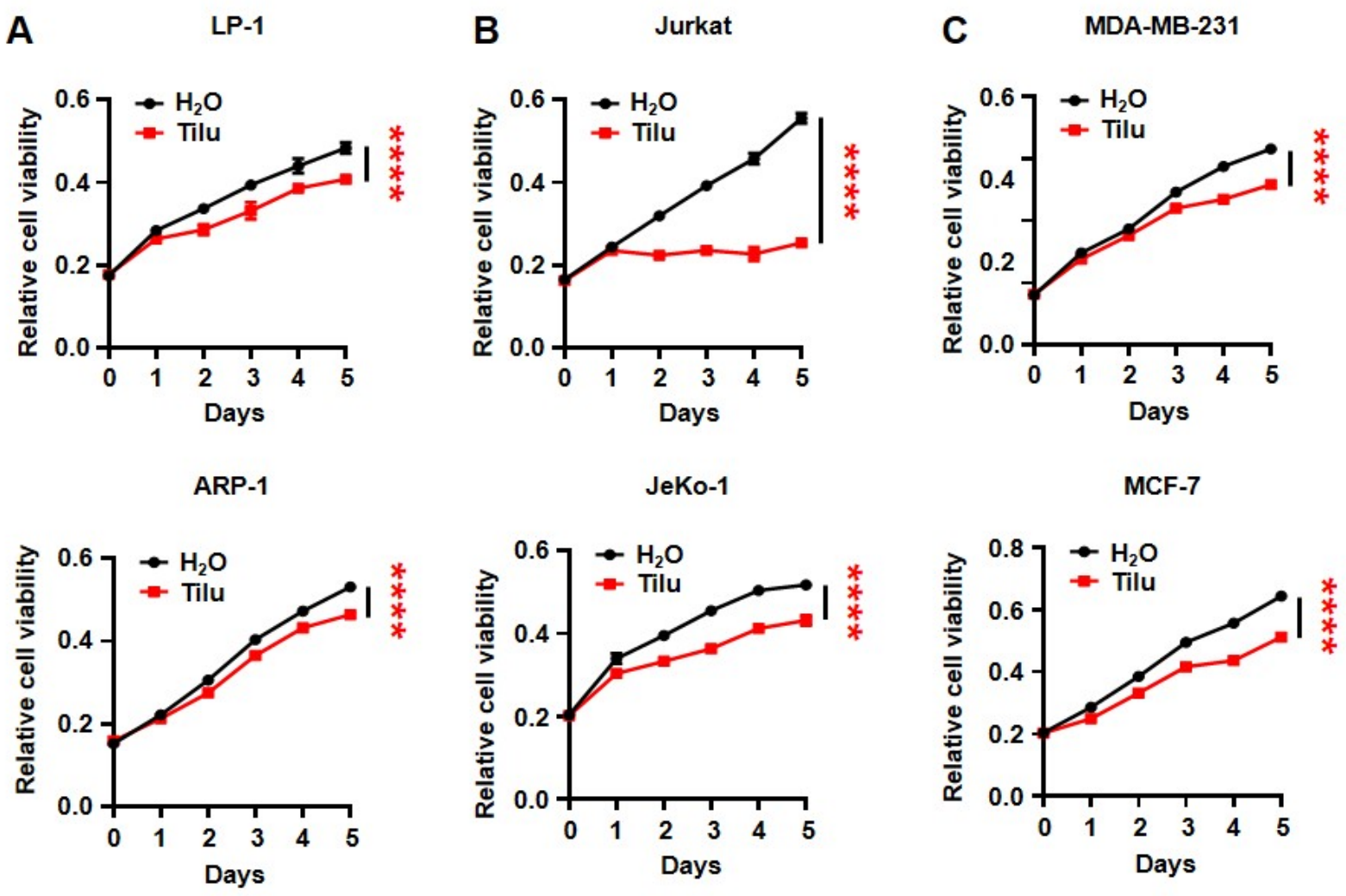

Figure S7. Tiludronate disodium inhibits the proliferation of myeloma, lymphoma and breast cancer cells. Related to Figure 6.

Proliferation of myeloma cell lines (LP-1 or ARP1) (**A**), lymphoma cell lines (Jurkat or JeKo-1) (**B**), or breast cancer cell lines (MDA-MB-231 or MCF-7) (**C**) treated with or without tiludronate disodium (400 μM) over time. Data are averages ± SD. ****$P < 0.0001$. P values were determined using two-way ANOVA.

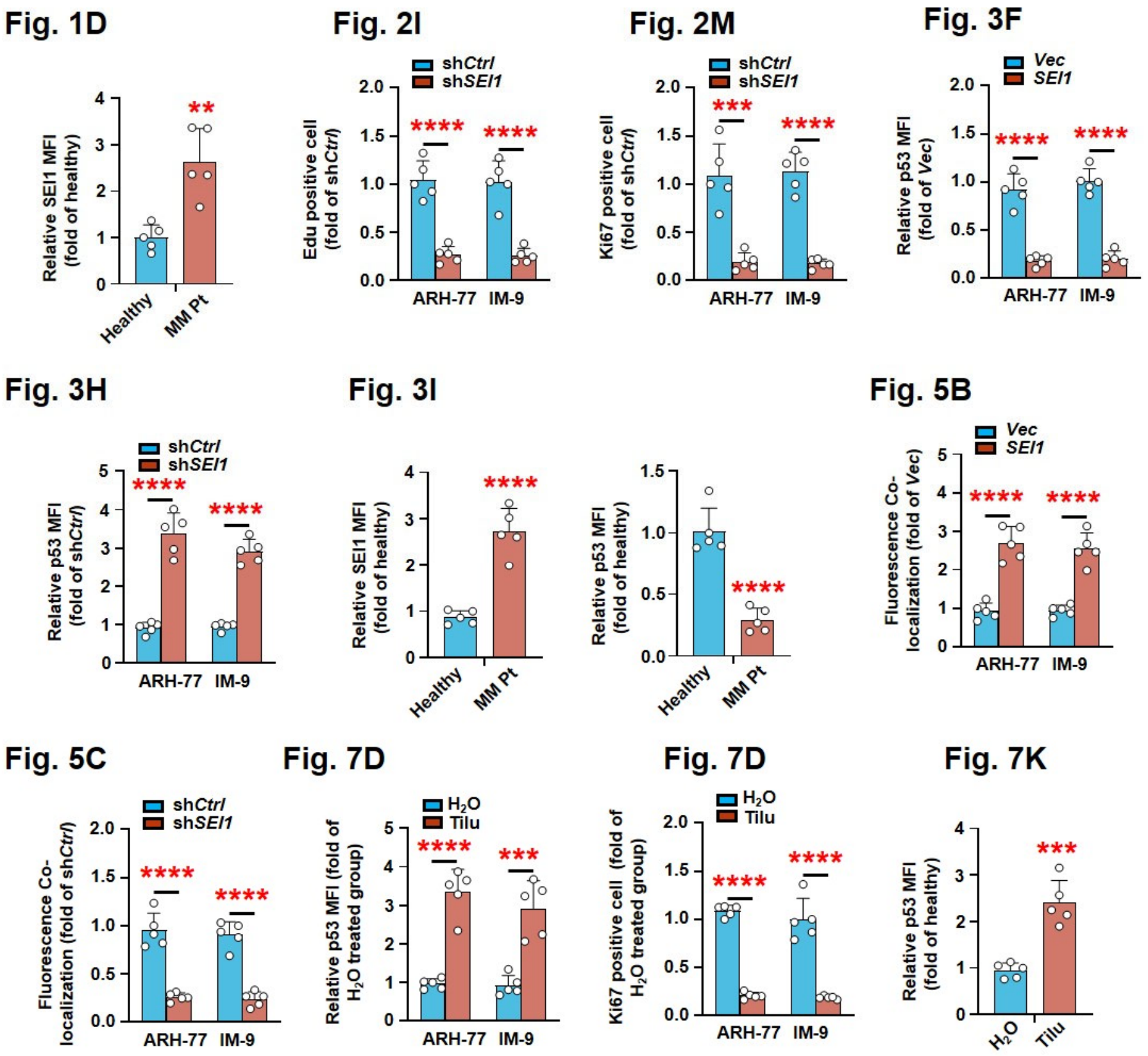

Figure S8. Quantitative analysis of immunofluorescence staining. Related to Figure 1, 2, 3, 5 and 7.

Mean fluorescence intensity (MFI) was measured with ImageJ software (v 2.1.0/1.53c). For each region of interest, the MFI per individual was derived by first calculating the quotient of the integrated fluorescence intensity from all captured fields divided by their combined area within that region. For each slide, the MFI of each respective marker was obtained from five randomly selected tumor areas. Data are averages ± SD. *P* values were determined by unpaired two-tailed *t* test. **$P < 0.01$, ***$P < 0.001$, ****$P < 0.0001$.

Table S2. Identification of SEI1 that binds to HDAC1 or FOXC1 in myeloma cells using mass spectrometry.

**Gene name**	**Peptides**
HDAC1	DGIDDESYEAIFKPVMSK
	KEVTEEEKTKEEKPEAK
	LHISPSNMTNQNTNEYLEK
	ISICSSDKR
	MTHNLLLNYGLYR
	SIRPDNMSEYSK
	VMTVSFHK
	YGEYFPGTGDLR
	YHSDDYIK
FOXC1	AYGPYTPQPQPK

Table S3. The primers used for amino deletion fragment of SEI1, SEI1 overexpression and shRNAs targeting primers.

Gene	Forward
SEI1-FL	CAAGCTTCGAATTCATGTACCCATACGATGTTCCAGATTACGCTATGCTGAGCAAGGGTCTGAA
SEI1-ΔCABD	CAAGCTTCGAATTCATGTACCCATACGATGTTCCAGATTACGCTACAGCGGTGGCACAGGCACCC
SEI1-ΔSD	AGATCCTGGCCCTAGCCCACCTGCAGCCCC
SEI1-ΔPBID	CCTGCTGGGCTCTGAGGGCCTCAAACCAGG
SEI1-ΔCT	GCCGGGCAAGCAGGCACTGGAGCGACCGCC
FOXC1-OE	CAAGCTTCGAATTCATGGACTACAAAGACGATGACGACAAGATGCAGGCGCGCTACTCCGTG
HDAC1-OE	CAAGCTTCGAATTCATGTACCCATACGATGTTCCAGATTACGCTATGGCGCAGACGCAGGG
shSEI1	CCGGTTTGACCTCTCAGTGCTCAAGCTCGAGCTTGAGCACTGAGAGGTCAAATTTTTG
**Gene**	**Reverse**
SEI1-FL	TAGAATTATCTAGATCAGCGCCCTGGCCCCGGCG
SEI1-ΔCABD	TAGAATTATCTAGATCAGCGCCCTGGCCCCGGCG
SEI1-ΔSD	GTGGGCTAGGGCCAGGATCTAGCCACCAGG
SEI1-ΔPBID	GGCCCTCAGAGCCCAGCAGGTCCAAGGCAC
SEI1-ΔCT	CCAGTGCCTGCTTGCCCGGCCCATCCTCAG
FOXC1-OE	CCCGGTAGAATTATCTAGATCAAAACTTGCTACAGTCGTAGACG
HDAC1-OE	TAGAATTATCTAGATCAGGCCAACTTGACCTCCTCC
shSEI1	AATTCAAAAATTTGACCTCTCAGTGCTCAAGCTCGAGCTTGAGCACTGAGAGGTCAAA

Table S4. Primers for real-time reverse transcription PCR analysis.

Gene	Forward	Reverse
GAPDH	ACAACTTTGGTATCGTGGAAGG	GCCATCACGCCACAGTTTC
SEI1	CAAGGGTCTGAAGCGGAAAC	GCTTGAGCACTGAGAGGTCAAA
p53	GAGGTTGGCTCTGACTGTACC	TCCGTCCCAGTAGATTACCAC
FOXC1	TGTTCGAGTCACAGAGGATCG	ACAGTCGTAGACGAAAGCTCC
p21	CGATGGAACTTCGACTTTGTCA	GCACAAGGGTACAAGACAGTG
PUMA	GACCTCAACGCACAGTACGAG	AGGAGTCCCATGATGAGATTGT
TIGAR	ATCCTGAAAGAAGCGGATCAAAA	AACTAAGACACTGGCTGCTAATC
HDAC1	CCGCATGACTCATAATTTGCTG	ATTGGCTTTGTGAGGGCGATA

Table S5. Primers used in ChIP-PCR.

Name	Forward	Reverse
ChIP-*p53*	CACCAGGTCGGCGAGAATCC	AGGCTCCTGGCACAAAGCTG

Table S6. Luciferase assay primers.

**Name**	**Forward**
Luc-p53-FL	CGTGCTAGCTCGAGAAGTTCTCAGGGATCCGACGCA
Luc-p53-Δ1	CTCCCTCATTTATTATCGAGCTCTTACTTG
Luc-p53-Δ2	GAAAGCAAATCTTATCAGAGTGATAAGGGTTG
**Name**	**Reverse**
Luc-p53-FL	GGAATGCCAAGCTTCCTGGACGGTGGCTCTAGACT
Luc-p53-Δ1	CGATAATAAATGAGGGAGACAGGTCTGAAG
Luc-p53-Δ2	GATAAGATTTGCTTTCAGTACATGGAAACGT

Data S1. Uncropped Western blot images.

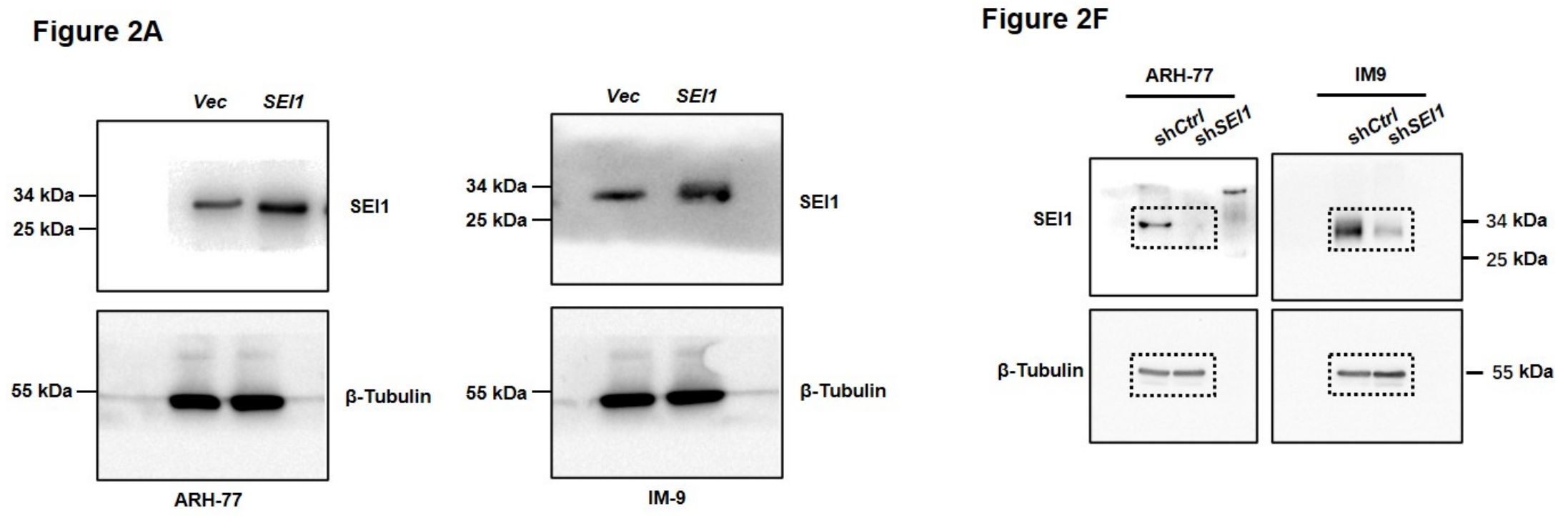

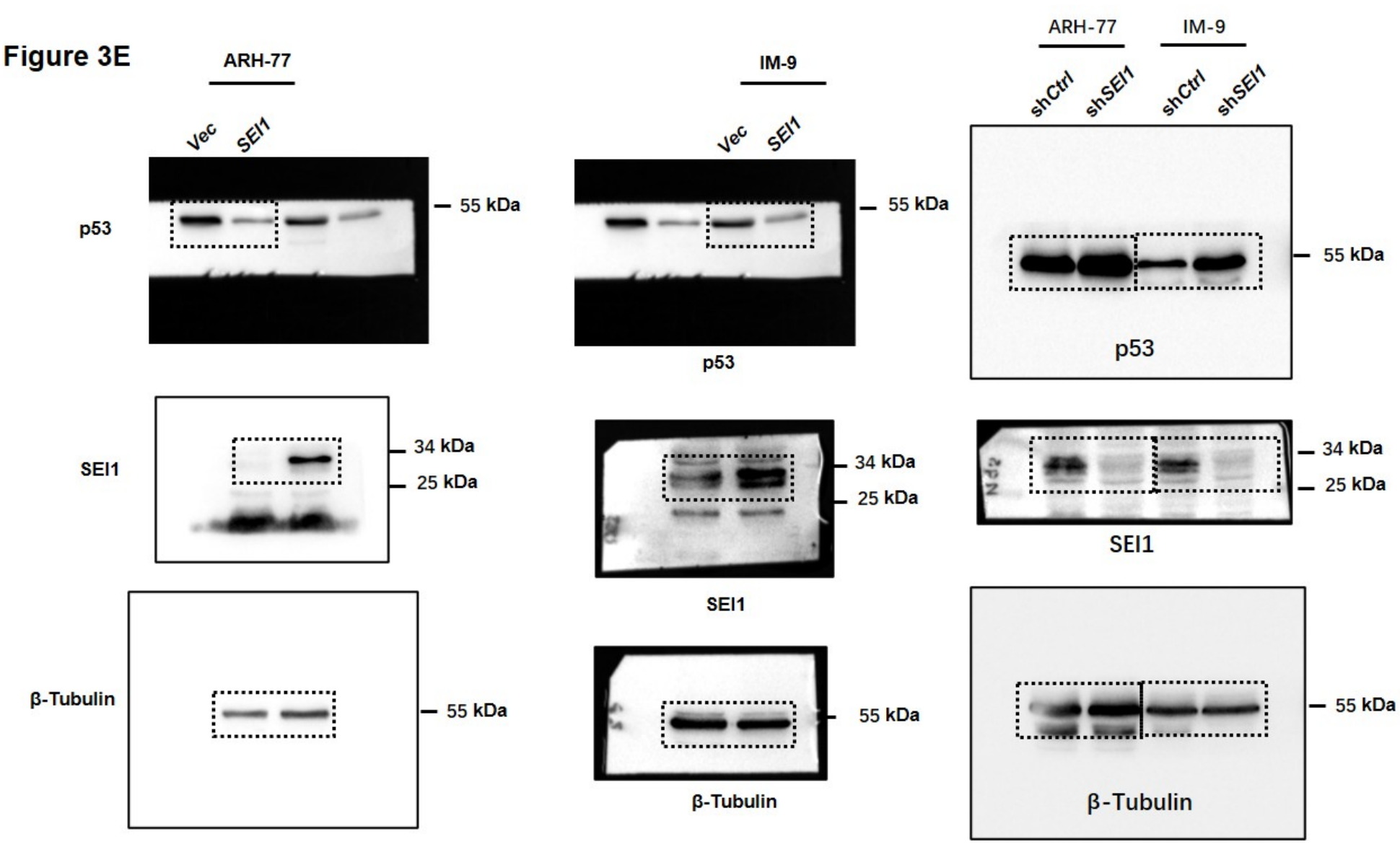

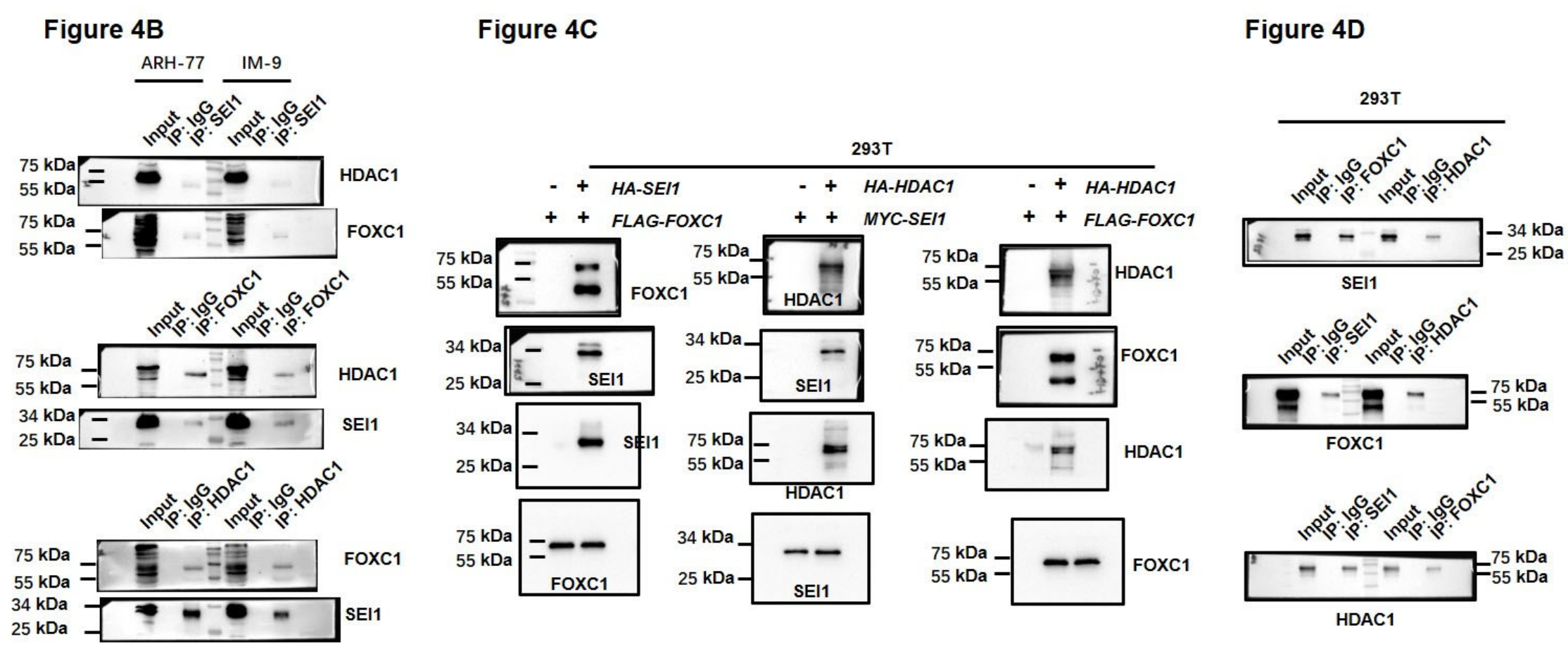

Figure 4G

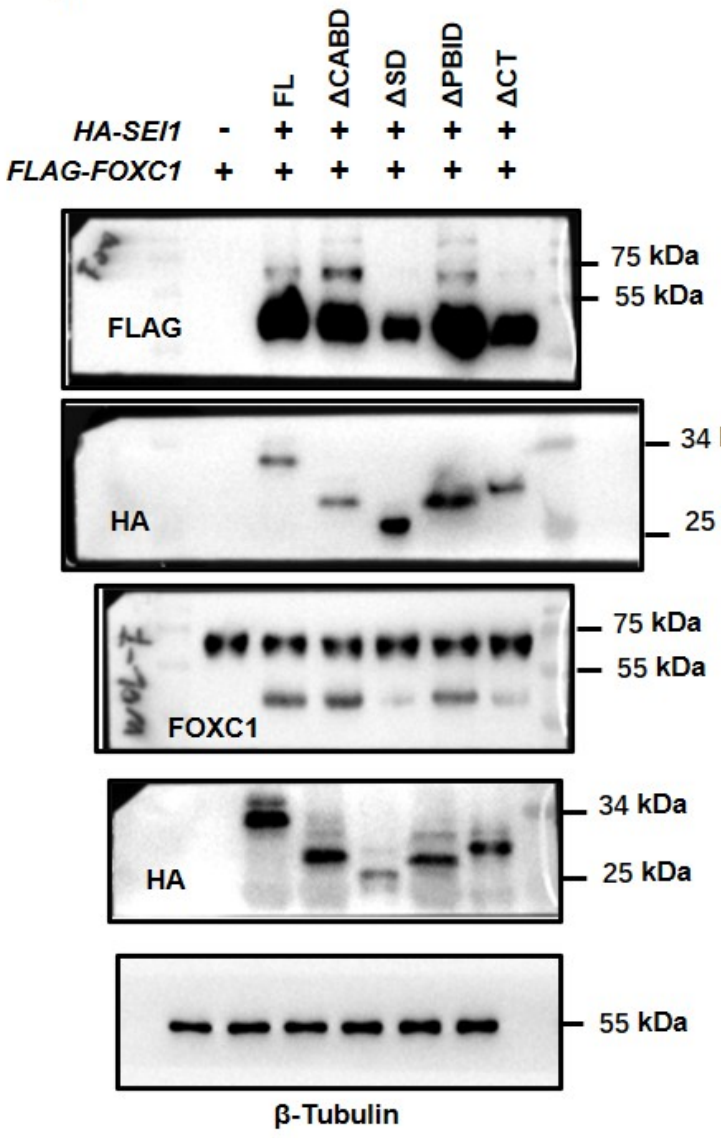

Figure 4H

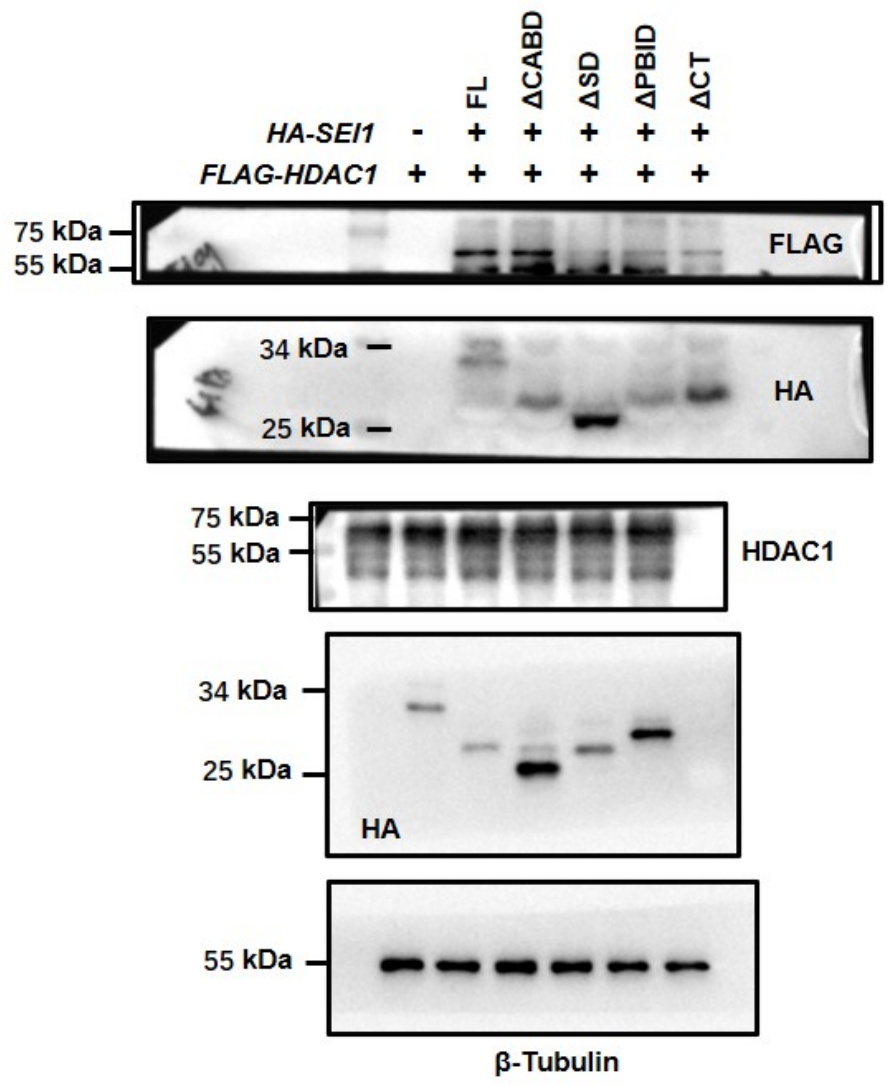

Figure 5A

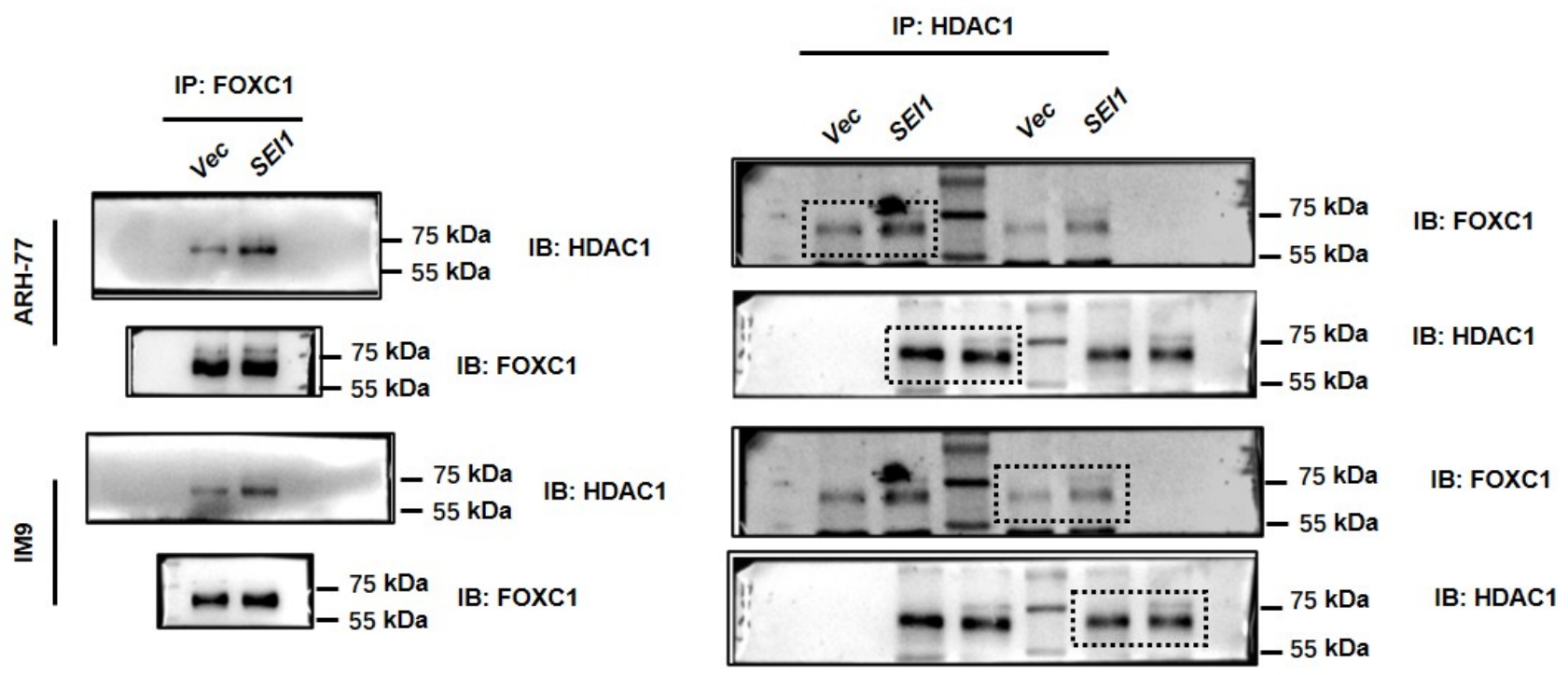

Figure 6E

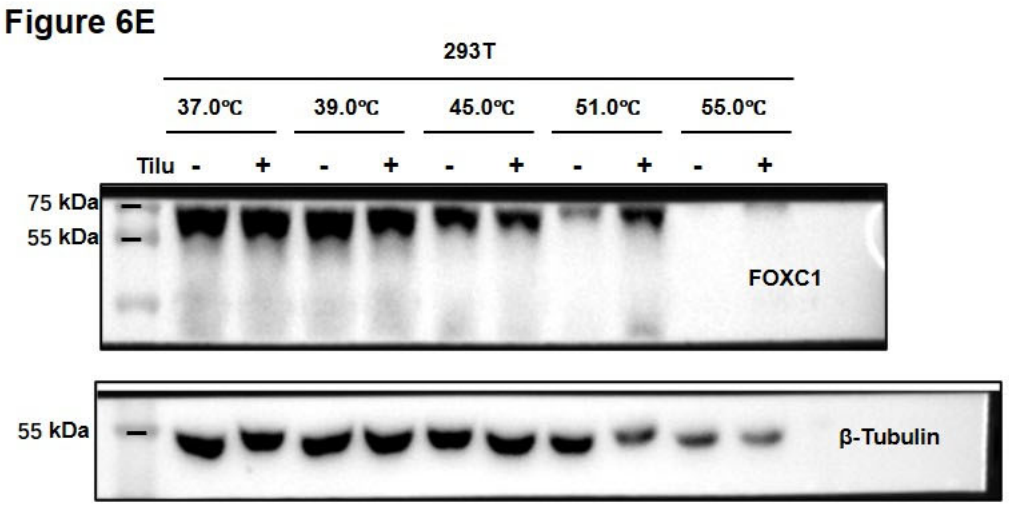

Figure 6G

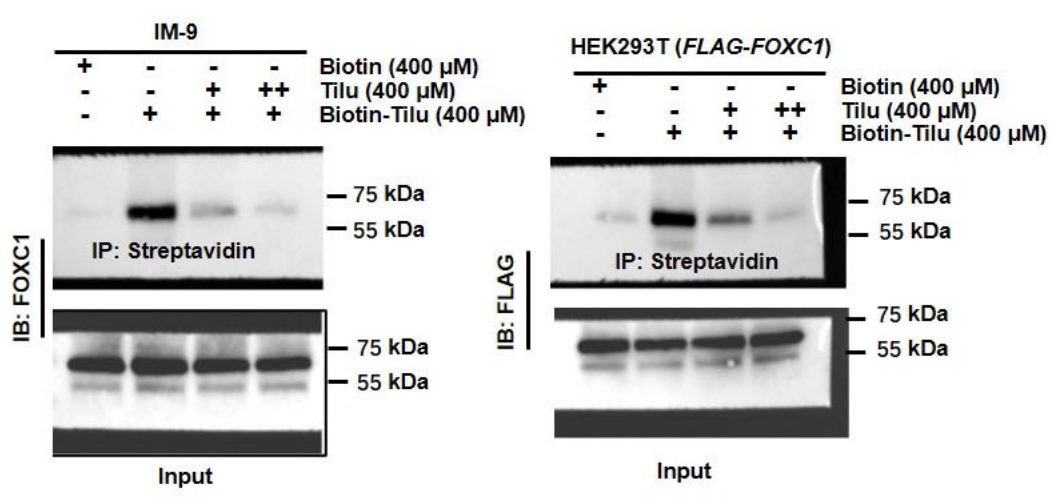

Figure 6I

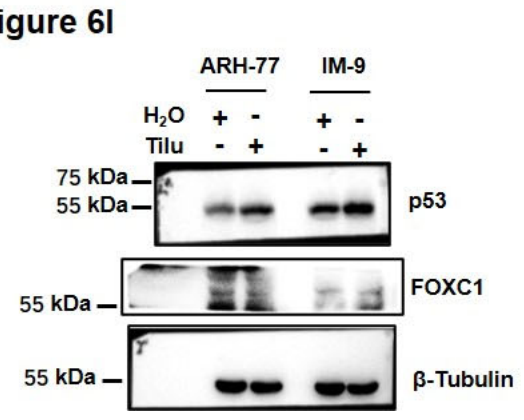

Figure 6J

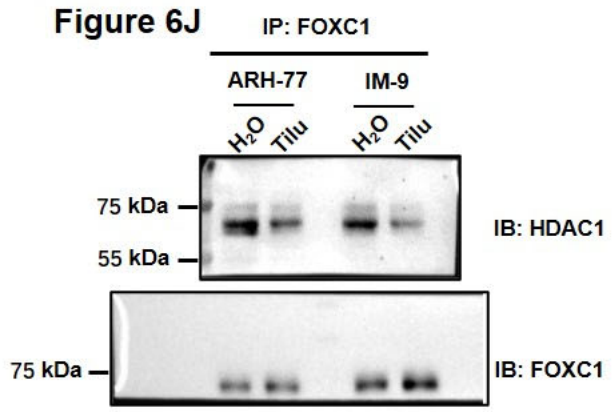

Figure S3

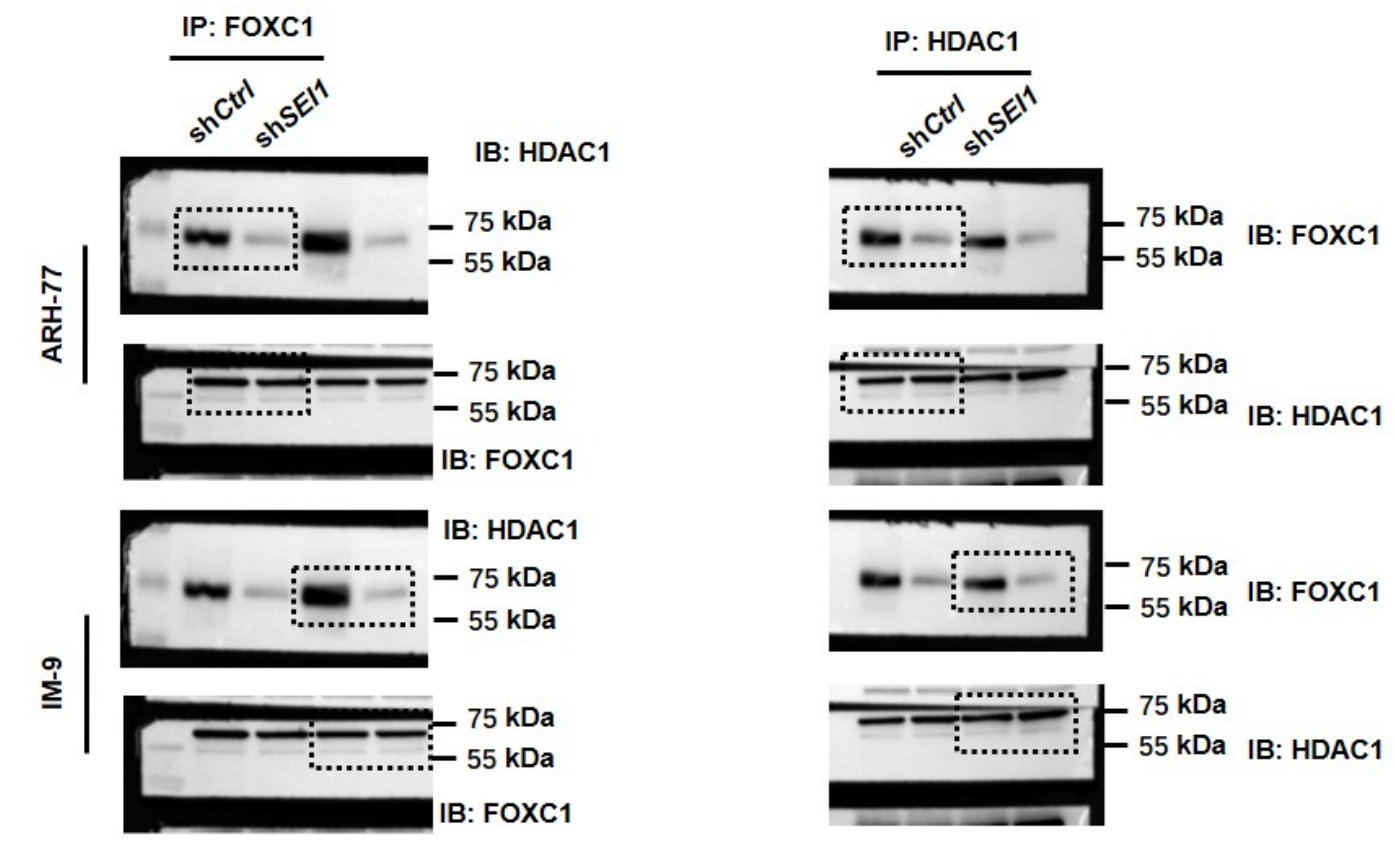

331
